# Position of rhodopsin photoisomerization on the disk surface confers variability to the rising phase of the single photon response in vertebrate rod photoreceptors

**DOI:** 10.1371/journal.pone.0240527

**Published:** 2020-10-14

**Authors:** Giovanni Caruso, Colin J. Klaus, Heidi E. Hamm, Vsevolod V. Gurevich, Clint L. Makino, Emmanuele DiBenedetto

**Affiliations:** 1 Italian National Research Council, Istituto di Scienze del Patrimonio Culturale, Roma, Italy; 2 The Mathematical Biosciences Institute, Ohio State University, Columbus, OH, United States of America; 3 Department of Pharmacology, Vanderbilt University Medical Center, Nashville, TN, United States of America; 4 Department of Physiology & Biophysics, Boston University School of Medicine, Boston, MA, United States of America; 5 Department of Mathematics, Vanderbilt University, Nashville, TN, United States of America; Carl von Ossietzky Universitat Oldenburg, GERMANY

## Abstract

Retinal rods function as accurate photon counters to provide for vision under very dim light. To do so, rods must generate highly amplified, reproducible responses to single photons, yet outer segment architecture and randomness in the location of rhodopsin photoisomerization on the surface of an internal disk introduce variability to the rising phase of the photon response. Soon after a photoisomerization at a disk rim, depletion of cGMP near the plasma membrane closes ion channels and hyperpolarizes the rod. But with a photoisomerization in the center of a disk, local depletion of cGMP is distant from the channels in the plasma membrane. Thus, channel closure is delayed by the time required for the reduction of cGMP concentration to reach the plasma membrane. Moreover, the local fall in cGMP dissipates over a larger volume before affecting the channels, so response amplitude is reduced. This source of variability increases with disk radius. Using a fully space-resolved biophysical model of rod phototransduction, we quantified the variability attributable to randomness in the location of photoisomerization as a function of disk structure. In mouse rods that have small disks bearing a single incisure, this variability was negligible in the absence of the incisure. Variability was increased slightly by the incisure, but randomness in the shutoff of rhodopsin emerged as the main source of single photon response variability at all but the earliest times. Variability arising from randomness in the transverse location of photoisomerization increased in magnitude and persisted over a longer period in the photon response of large salamander rods. A symmetric arrangement of multiple incisures in the disks of salamander rods greatly reduced this variability during the rising phase, but the incisures had the opposite effect on variability arising from randomness in rhodopsin shutoff at later times.

## Introduction

**Fig 1:**
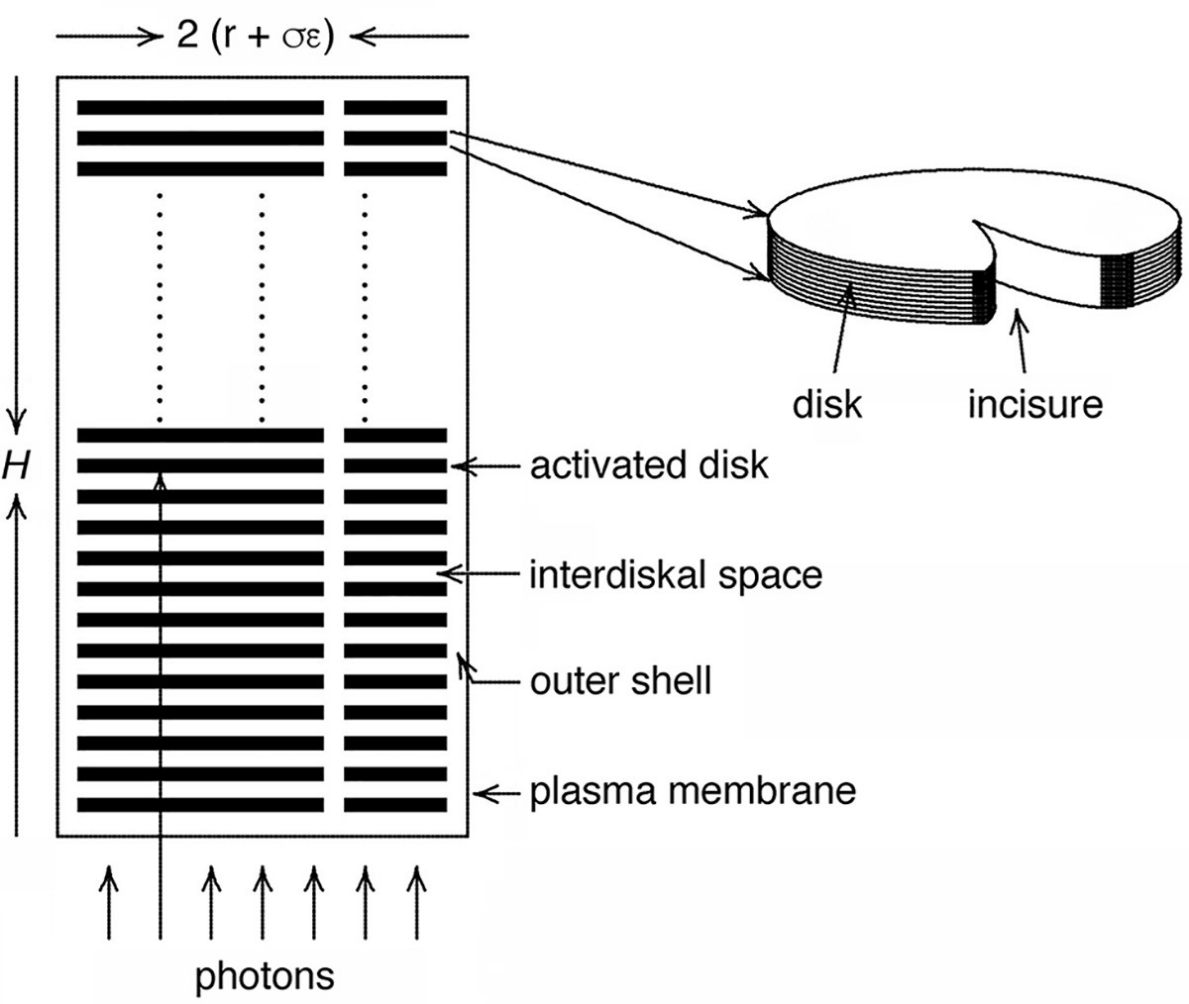
Disk barriers to the axial diffusion of cGMP and Ca^2+^ in the rod outer segment. Membranous disks of radius r segment the cytoplasm into layers of interdiskal space, 14.5 nm in thickness. Layers are interconnected by an outer shell of thickness σε that separates the disks from the plasma membrane, as well as by the single incisure in each mouse rod disk (as shown) and the multiple incisures in each salamander rod disk. In mouse, the outer segment diameter = 2(r + σε) is ~1.4 μm [5], whereas in salamander, it is typically 10–12 μm [6, 7], but it can be as large as 13 μm [8].

In the vertebrate retina, rod photoreceptors provide visual input under very dim light. The conversion of light into an electrical signal occurs within a specialized cilium called the outer segment. Therein hundreds to thousands of flattened disks whose membranous surfaces are densely packed with rhodopsin, capture photons efficiently (**Fig 1**). Photoisomerization of rhodopsin initiates a G protein cascade restricted to one face of a disk, that culminates in the hydrolysis of cGMP by activated phosphodiesterase (PDE*). The ensuing fall in cGMP concentration in the cytosolic volume between disks that propagates to their rims closes cyclic nucleotide gated (CNG) channels in the plasma membrane (reviewed in [1, 2]). The subsequent reduction in an inward current carried by Na^+^ and Ca^2+^ through the channel hyperpolarizes the membrane. The single photon response (SPR) builds relatively slowly because amplification in this cascade takes time, but downstream rod "ON" bipolar cells improve upon temporal resolution by signaling on a faster time scale [3, 4]. Thus, it appears that for the single photon response, or more generally for very dim illumination levels, the rising phase of the rod response is most critical for vision.

The SPR must be reproducible to carry meaningful information about light intensity. Rhodopsin is activated by photoisomerization of its covalently attached chromophore, 11-*cis* retinal, which is converted to all-*trans* retinal. A major source of variability affecting the peak and recovery phase of the SPR originates from randomness in the inactivation of photoexcited rhodopsin, R* [9–11]. R* is phosphorylated (up to 6 or 7 times in different species) with a variable delay separating the addition of each phosphate, and by the subsequent binding of arrestin-1 (a.k.a. visual or rod arrestin), which is dependent upon the number of phosphates added [12]. Progressive phosphorylation reduces R* activity [13, 14], but arrestin-1 binding is necessary for complete quench [15]. However, randomness in R* shutoff does not greatly affect the early rising phase of the response since it is unlikely that the first phosphorylation of R* will have even occurred during that time [16, 17]. A potentially important source of variability during this period arises from randomness in the location of the rhodopsin photoisomerization on the disk [18, 19]. Rhodopsin, visual G protein transducin, and PDE are anchored in the membranes of internal disks in the rod outer segment. Following photon absorption by a rhodopsin molecule at the disk rim, activation of the first PDE produces a local depletion of cGMP in the cytosol in proximity to the CNG channels localized in the plasma membrane. Channel closure is relatively fast and vigorous. In contrast, the response to a photoisomerization at the center of the disk, i.e. farthest from the channels, is delayed as the local reduction in cGMP level in the cytoplasm between adjacent disks spreads and dissipates radially and through incisures axially, before finally impacting the CNG channels [20]. The delay will be brief if disk radius is small, but will be extended by an increase in disk size. Closure of the CNG channels reduces the concentration of Ca^2+^ in the cytoplasm. The reduction of Ca^2+^ leads to its replacement with Mg^2+^ on GCAPs, converting them from inhibitors to activators of guanylyl cyclase, which replenishes cGMP, leading to the opening of CNG channels, thereby terminating the signal.

Randomness in the spatiotemporal fall in cGMP due to R* location wanes in importance over time as PDE*s spread across the disk surface and cGMP diffuses throughout the outer segment, eventually yielding to randomness in R* inactivation as the main source of SPR variability. Despite the importance of the rising phase of the SPR to visual signaling, little is known about the relative contribution of each source of variability and about the time when the transition in dominance occurs. Here, we explore these issues with a fully space-resolved biophysical model of phototransduction that accounts for radial as well as longitudinal diffusion of cGMP and Ca^2+^. We used the model to evaluate different sources of variability, in isolation or in combinations, of the rising phase of the photon response. Since variability due to the location of photoisomerization should depend on disk size, we systematically varied disk radius and also applied the model to rods of salamander and mouse, whose disk radii differ by almost an order of magnitude [5, 8]. Disks in mouse rods have a single incisure [5, 21], whereas disks in salamander rods are partitioned by numerous incisures [8]. Incisures affect the movement of rhodopsin, transducin, and PDE on the disk membrane surface and also promote the axial diffusion of cGMP and Ca^2+^ in the cytosol, so some simulations were performed for both salamander and mouse rods in which the incisures were “removed” to test their function.

## Methods

The fully space-resolved model of phototransduction was used with different parameter sets for salamander rods and for mouse rods. The model described previously [11, 18, 20, 22, 23] and parameter sets, similar to those in [19, 20, 22, 24] but with minor computational adjustments, are included as **S1 Appendix**, for completeness. These parameters and the formalism describe the independent activation of individual catalytic subunits of the PDE dimer by single transducins. The requirement for two transducins to simultaneously bind the PDE dimer before there is any significant activation [25] was tested in additional simulations. Our approach of using the heat equation as the solution for the spread of PDE activation across the disk surface [26] applies to both scenarios, as is shown below and in **S1 File**. The results presented below were obtained from the finite element code in Matlab format, accessible on Github (doi: 10.5281/zenodo.3334503), with the parameters specified in **S1 Appendix**. Some parameter substitutions were made for selected simulations to test specific hypotheses on the effect of incisures, disk radius and diffusion coefficient, as described in the text.

Each simulation was initiated by the creation of an R* in the central disk of the outer segment, so any variability arising from differences in SPRs elicited at the proximal and distal disks [27–30] was not tested. The site of rhodopsin activation was fixed on the disk surface at one of three specified positions for some simulations or at a random location to test for a role of its position on SPR variability. In the homogenized model, the outer segment was subdivided into three domains: the outer shell (treated as a cylindrical surface), the interior volume (a cylinder) and the special disk (disk containing the photoisomerized rhodopsin). The mesh of the outer shell used quadrilateral elements, the mesh of the interior volume employed prisms and the mesh of the special disk employed triangular elements. Linear shape functions mapped the unknowns inside each element in terms of their nodal values. A refinement along the axial direction around the activated disk level was adopted to account for the large axial gradients occurring there. A standard iso-parametric map was used to map the elements in the actual geometry into corresponding reference elements. Boundary conditions were enforced at each end of the interior volume and of the cylindrical outer shell and it was assumed that there was no axial flux of transduction proteins.

Shutoff of R* activity proceeded with step-wise decrements in activity, each activity state having its own duration, according to the solution of a continuous time Markov chain [11]. For simulations probing the randomness in R* shutoff, the duration of each R* state had an exponential distribution with a mean found from curve fitting (see **S2, S4 Tables** in **S1 Appendix**). In all other simulations, R* shutoff was deterministic, with the duration of each activity state assigned to the mean value for that state. For salamander, the average durations of the phosphorylated states were (s): 0.0833, 0.1000, 0.1250, 0.0625, 0.0714, 0.0833, and 0.1000, with an average R* lifetime of 0.4 s. For mouse, the average durations were (s): 0.0159, 0.0190, 0.0238, 0.0109, 0.0123, 0.0142, and 0.0167, with an average R* lifetime of 0.08 s.

The behavior of the CNG channels was modeled as a population. This decision was justified by the channel's low unitary conductance under physiological conditions, coupled with their high density (reviewed in [31]) and uniform axial distribution in the plasma membrane of the outer segment [32].

One thousand simulations were run for each set of parameters and conditions, after which the coefficient of variation (CV) was computed as the ratio of the standard deviation to the mean for the total influx of current into the rod outer segment (j_tot_), carried by the CNG channels and the Na^+^/K^+^, Ca^2+^ exchanger. As will be discussed below, when assessing the variability at early times in the photon response, it was also useful to compute CV for the relative drop in current after photon absorption, I(t) = 1- j_tot_/ j_dark_, where j_dark_ is the j_tot_ value in darkness.

## Results

### Effect of activation site on SPR variability

The first assessment of the impact of the location of rhodopsin activation on the SPR of a large salamander rod was made by comparing deterministic simulations for an R* at each of three fixed positions on the disk surface: center of the disk, halfway between disk center and the rim, or at the rim. The spatiotemporal spread of transducin/PDE activation on the disk surface was modeled according to the diffusion of heat across a surface [26]. This approach yielded the average response to the photoisomerization at each location so that the effect of photoisomerization position could be assessed in isolation. Although randomness in the spatiotemporal pattern of PDE activation across the disk would confer additional variability in the rising phase of the SPR, its contribution to CV appears to be marginal [19]. The basal PDE activity was modeled as spatially uniform to remove confounding effects introduced by the discrete, spontaneous PDE activations that generate the continuous noise, cf. [33]. Disks in amphibian rods have multiple incisures that penetrate from the disk rim towards its center [8, 34, 35]. For simplicity and to isolate the effect of R* position, the incisures were "removed" from the disks for the initial simulations.

**Fig 2:**
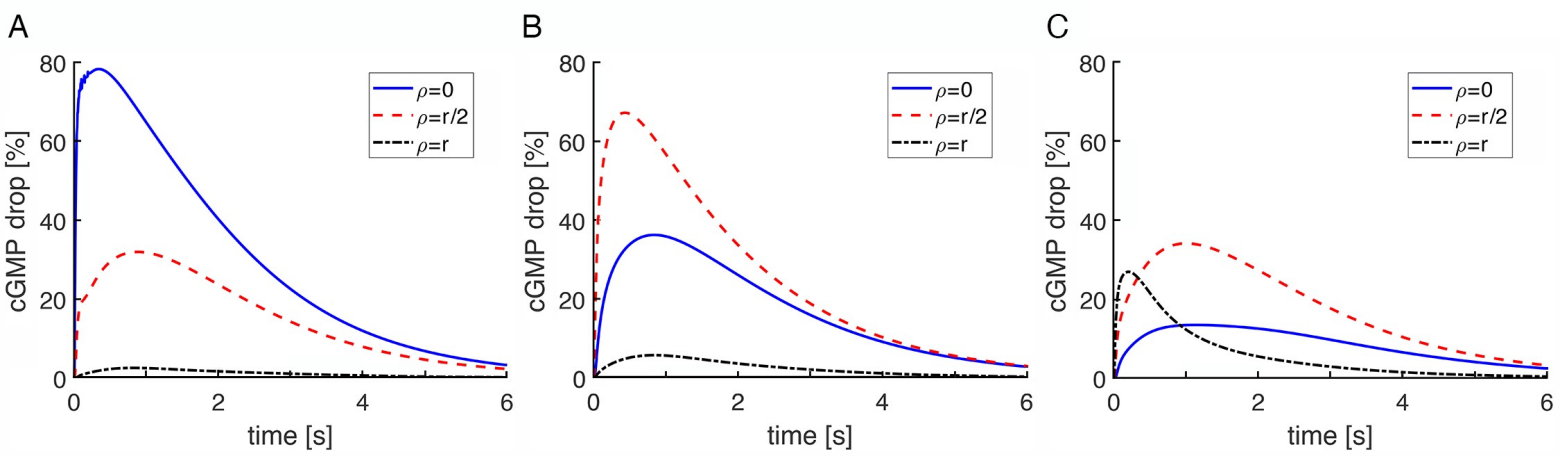
Dependence of spatiotemporal changes in cGMP on R* location in a salamander rod. At time zero, a photoisomerization was placed in the center of the disk (**A**), halfway between the center and the rim (**B**), or near the disk rim (**C**) in a rod lacking incisures. The relative drop in [cGMP] was computed at three interdiskal locations: next to the center of the activated disk (ρ = 0), halfway between the center of the disk and the disk rim (ρ = r/2), and near the disk rim (ρ = r). For (**A**), the changes in [cGMP] were radially symmetric, whereas for (**B** and **C**), the changes in [cGMP] at ρ = r/2 and ρ = r are shown only for the locations closest to the photoisomerization. Disk radius, r, was 5.5 μm and the diffusion coefficient for cGMP, D_cG_, was 160 μm^2^/s.

Photoisomerization of a rhodopsin on the disk gave rise to striking radial gradients in cGMP that evolved and persisted throughout the duration of the photocurrent response (**Fig 2**). The form of the gradient varied markedly with the location of the photoisomerization. After a photoisomerization in the center of the disk, cGMP levels dropped dramatically in the overlying cytoplasm as a PDE was activated (**Fig 2A**). Over time, the decrement in cGMP spread radially, as cGMP diffused inward from the periphery and PDE*s on the disk surface diffused to increasing distances from the disk center. However, levels of cGMP near the plasma membrane never dropped very low, because of replenishment by longitudinal diffusion. In addition, CNG channel closure caused an initially local fall in [Ca^2+^] that stimulated cGMP synthesis by guanylate cyclase [1, 2], which was taken to be uniformly distributed on the disk membranes. This radial profile of cGMP is in general agreement with the previous results obtained with the fully space-resolved model [18, 20].

With a photoisomerization halfway between the center of the disk and the rim, the site of greatest cGMP depletion moved outward (**Fig 2B**). The change in cGMP at the nearest plasma membrane was greater, but followed a similar time course as before. A photoisomerization at the disk rim caused the largest and most rapid drop in cGMP at the plasma membrane near the site of photoisomerization (**Fig 2C**). But as cGMP levels began to recover there, cGMP fell to an even lower minimum in the interdiskal space halfway to the disk center. Later, as cGMP levels near the site of photoisomerization recovered further, the cGMP levels near the disk center continued to fall, dropping below the levels at the rim but never dropping below the levels halfway between the center and the rim.

**Fig 3:**
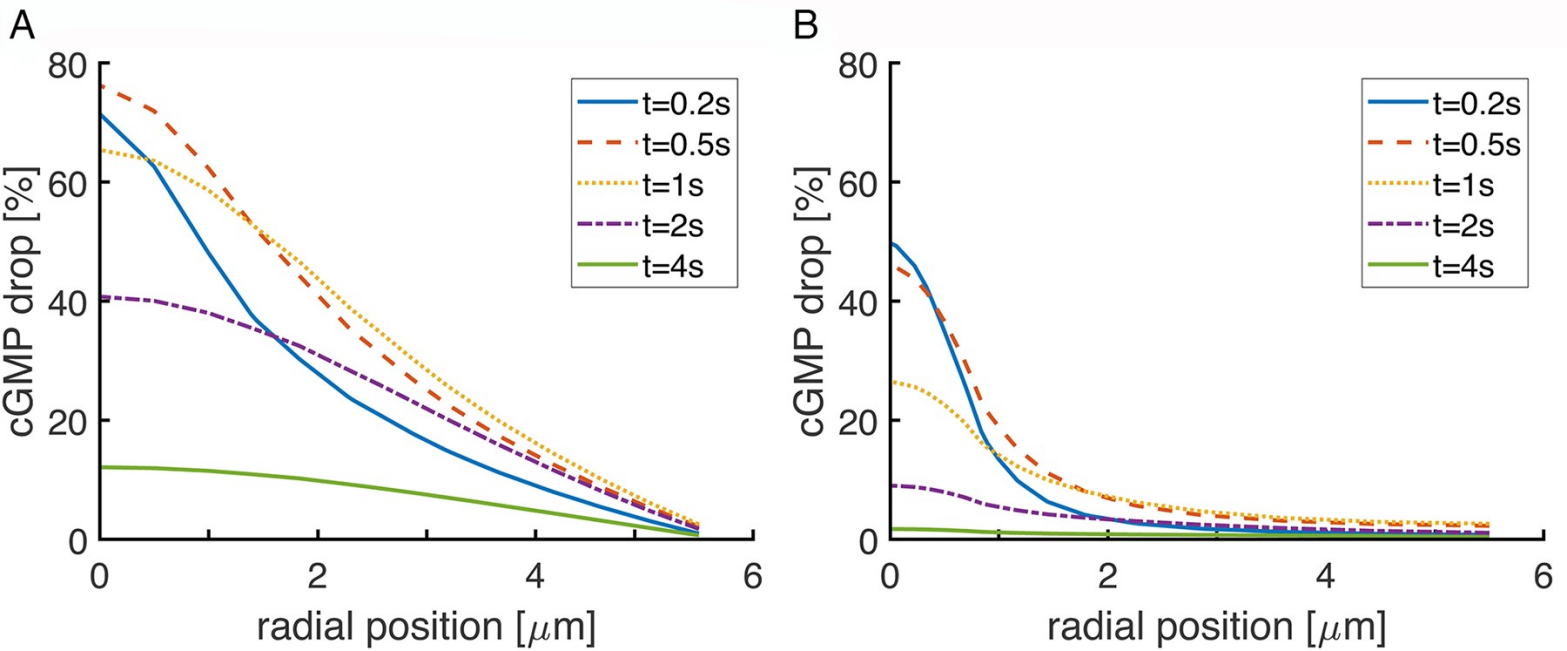
Reduced drop in cGMP and faster recovery with incisures in a salamander rod. The drop in cGMP is shown for the interdiskal space at the level of the active disk. The simulations were deterministic with the photoisomerization located in the center of the disk for a rod lacking incisures (**A**) and for a rod with 23 incisures evenly spread around the disk rim (**B**). Disk radius was 5.5 μm. Incisures extended 4.64 μm inward from the disk rim and were aligned in all disks.

**Fig 4:**
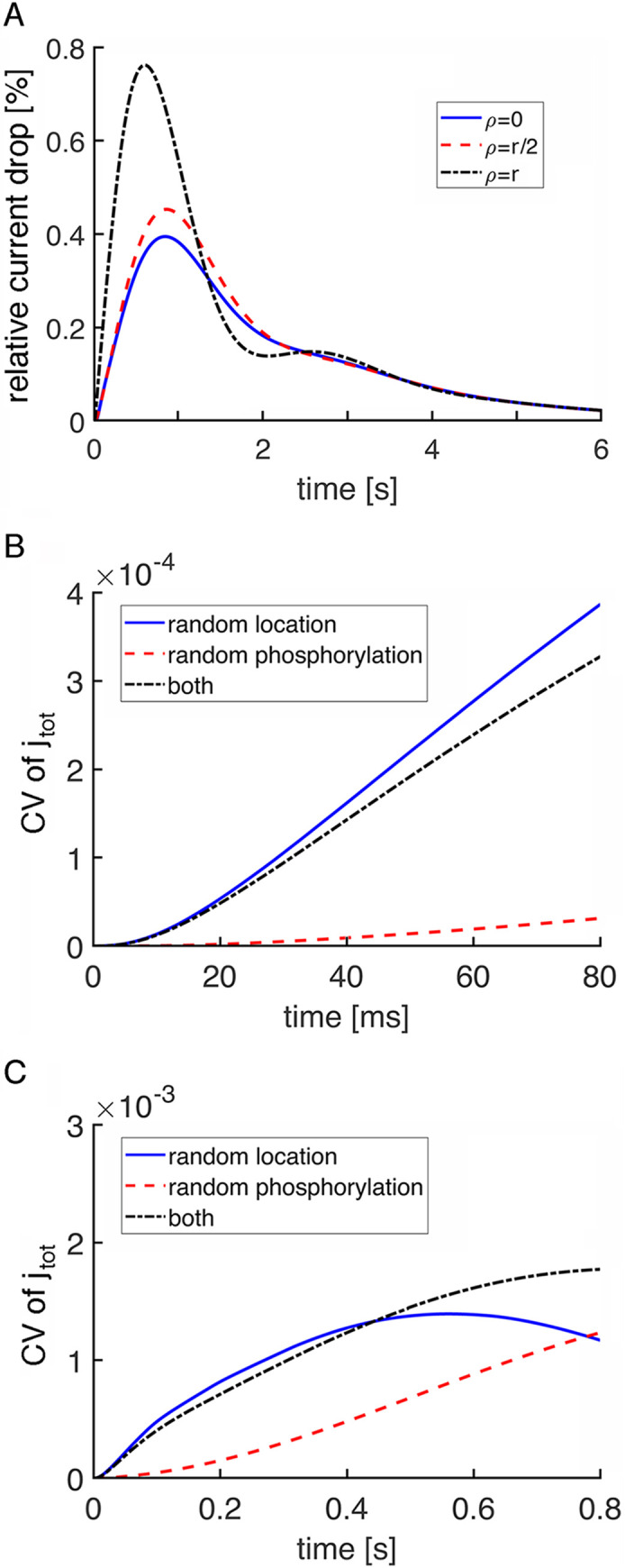
Larger, faster SPR elicited by R* at the disk rim of a salamander rod. (**A**) The three traces show %I(t) in a rod for R* positioned at: the disk center (ρ = 0), the disk rim (ρ = r), or an intermediate position (ρ = r/2), where r was 5.5 μm. The simulations were deterministic with the spread of transducin/PDE activation across the disk following the diffusion of heat on a surface and with R* and transducin/PDE activities shutting off over exponential time courses. The incisures were removed from all disks. (**B**) In stochastic simulations, randomness in R* location dominated CV of total current, j_tot_(t), at early times. (**C**) Later, towards the end of the rising phase of the SPR, CV due to randomness in R* location declined to match the CV due to randomness in R* shutoff.

The spatiotemporal profiles of cGMP in three dimensions within the outer segment, which were radially asymmetric except for the photoisomerization at the disk center (**Fig 3A**), were then used to compute the photocurrent responses. The two responses to rhodopsin photoisomerizations in the interior of the disk were similar but lagged that after a photoisomerization at the disk rim and were reduced in peak amplitude by nearly two-fold (**Fig 4A**). These differences in SPR amplitude and kinetics were more pronounced, but were otherwise in accordance with those reported previously [18]. In the present study, the steep, initial recovery in the SPR arising from a photoisomerization at the rim was caused by the greater local fall in Ca^2+^ and more powerful stimulation of guanylate cyclase near the plasma membrane. After ~ 2 s, guanylate cyclase activity was subsiding, but at axial distances several μm from the active disk, cGMP synthesis near the outer shell actually exceeded diffusion of cGMP towards the inactivating PDE*s and cGMP levels climbed to levels higher than normally present in darkness. Eventually, rising Ca^2+^ levels inhibited this exuberant guanylate cyclase activity, and the presence of residual PDE*s gave rise to a damped oscillation in the recovery phase. Differences in the SPRs were still present late in the recovery phase, eventually converging after about 3.5 s. These dramatic differences demonstrate that randomness in the R* location contribute to variability over the early part of the SPR.

The relative contribution of R* location to variability in the rising phase of the SPR was compared to the contribution of randomness in R* phosphorylation by carrying out three sets of stochastic simulations. For set 1, R* position was randomly chosen in each trial with an even distribution over the entire disk surface while R* inactivated with a deterministic time course using average values for the duration of each phosphorylation state. For set 2, R* position was fixed at the center of the disk, while the intervals between phosphorylation steps were chosen randomly from an exponential distribution with average equal to the average duration of each step. The average time for the first phosphorylation in the salamander rod was set to 83 ms, obtained by curve fitting of an experimental SPR (**S1 Fig** in **S1 Appendix**). R* catalytic activity was decremented with the addition of each phosphate as described in [11, 19] (see **S2 Table** in **S1 Appendix**). For set 3, R* location and phosphorylation of R* were both randomized. One thousand simulations of total current, j_tot_, were computed for each set of conditions. Variability was assessed as CV of the total current, the ratio of the standard deviation of j_tot_ to the mean of j_tot_.

Randomness in R* location dominated CV during the rising phase of the SPR (**Fig 4B**). For part of this period, CV was actually lower when R* location and R* shutoff were both randomized, indicating that by reducing R* catalytic activity, early phosphorylations improved the reproducibility of the response to an R* acting at different sites. Randomness in R* shutoff became equally important near the peak of the SPR (**Fig 4C**) and superseded randomness in R* location thereafter as the major source of variability throughout the recovery phase.

### Incisures dampen the variability due to photoisomerization location

**Fig 5:**
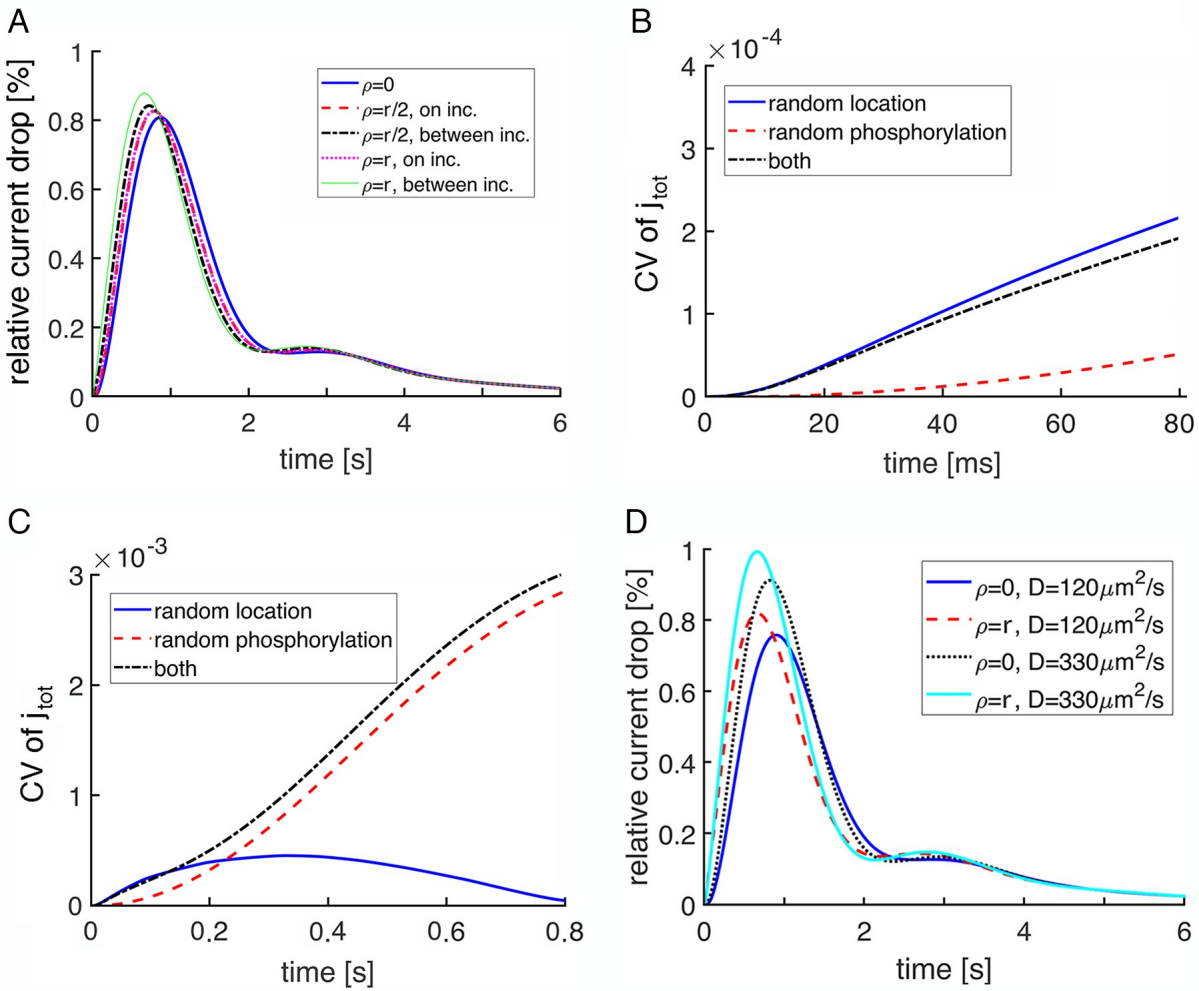
Early SPR variability reduced by multiple incisures in a salamander rod. Each disk had 23 incisures distributed evenly around its perimeter. Disk radius was 5.5 μm and each incisure extended 4.64 μm inward from the disk rim. Incisures were aligned in consecutive disks. (**A**) Deterministic simulations of %I(t) were computed for the R* located: at the disk center (ρ = 0), halfway to the rim (ρ = r/2) either midway between incisures or adjacent to one of them, and at the rim (ρ = r) either between incisures or adjacent to one of them. D_cG_ was 160 μm^2^/s. (**B**) CV for j_tot_ was dominated by randomness in R* location during the initial rising phase of the SPR, based on stochastic simulations. R* was located in the middle of the disk for trials with random R* phosphorylation. (**C**) CV due to randomness in R* location subsided over time and was eventually surpassed by CV due to randomness in R* phosphorylation at ~0.25 time to the peak of the SPR. (**D**) Deterministic simulations comparing the %I(t) after a photoisomerization at the disk center or at the rim halfway between two adjacent incisures for two different D_cG_ values.

A salamander rod disk bears numerous incisures that penetrate deeply into its interior [8]. Given that incisures facilitate the axial diffusion of cGMP and Ca^2+^ in the aqueous cytosol, they enable a greater number of cGMP molecules to be hydrolyzed, spread the change in cGMP over a greater axial distance, and reduce the cGMP drop at the level of the activated disk (**Fig 3B**). Incisures also obstruct the diffusion of membrane proteins on the disk surface. Their influence over the SPR therefore affects both its amplitude and variability [18, 19]. The interaction of incisures with randomness in the location of photoisomerization was explored with deterministic simulations of the relative drop in current, %I(t) (**Fig 5A**), and with calculations of CV from stochastic simulations of total current, j_tot_ (**Fig 5B and 5C**) in salamander rods with 23 radial incisures arranged symmetrically in the disk. For the deterministic simulations, we considered five R* positions: center of the disk, halfway to the rim and midway between two incisures, halfway to the rim and adjacent to an incisure, at the rim and midway between two incisures, and at the rim and adjacent to an incisure.

The presence of incisures in the disk augmented the amplitudes of SPRs due to activations at all locations and reduced the amplitude difference between the response to activation at disk center and that at the disk rim from about 2-fold (**Fig 4A**) to less than 1.1-fold in deterministic simulations (**Fig 5A**). When the activation occurred next to an incisure, the drop in cGMP had a greater axial spread and the rate of change at the plasma membrane was slowed. As a result, the SPR was intermediate between that elicited by photoisomerization at disk center and that elicited at the rim between incisures, and became largely independent of radial R* location. The SPR was still largest for an activation at the rim, a result at variance with [18], where incisures caused a reversal so that the SPR became largest for an activation at the disk center. The basis was traced to the more rapid diffusion coefficient for PDE used in the earlier study; when the same value was adopted here, the reversal also occurred (results not shown). Greater access of PDE* to cGMP in the outer shell with incisures caused SPRs due to R*s at all locations on the disk to more closely resemble the response to an R* at the rim not only in size, but also in exhibiting the damped oscillation in the recovery phase.

Stochastic simulations revealed that incisures substantially decreased the CV due to randomness in R* location (**Fig 5B and 5C**) and had the opposite effect on CV due to randomness in R* phosphorylation. The former occurred because a symmetric pattern of long incisures tended to restrict the spread of activation to sectors of uniform size on the disk surface. The latter was caused by the incisures improving the axial diffusion of second messengers inside the rod and accordingly, reducing the local depletion of cGMP that would lower CV due to randomness in the rhodopsin phosphorylation, as described previously [18, 19]. There was also a more rapid decline in CV due to R* location such that CV due to R* phosphorylation matched it in importance at ~200 ms and far surpassed it by the peak of the SPR (**Fig 5C**).

A faster diffusion rate for cGMP within the outer segment supported larger SPRs with faster kinetics over the first ~4 s (**Fig 5D**). Slower diffusion of cGMP had the opposite effects. However, the SPRs produced by R*s in the disk center and at the disk rim were only marginally more similar in amplitude with the faster diffusion rate and the oscillation in the recovery became slightly more pronounced.

### Negligible effects of activation location in mouse rods

**Fig 6:**
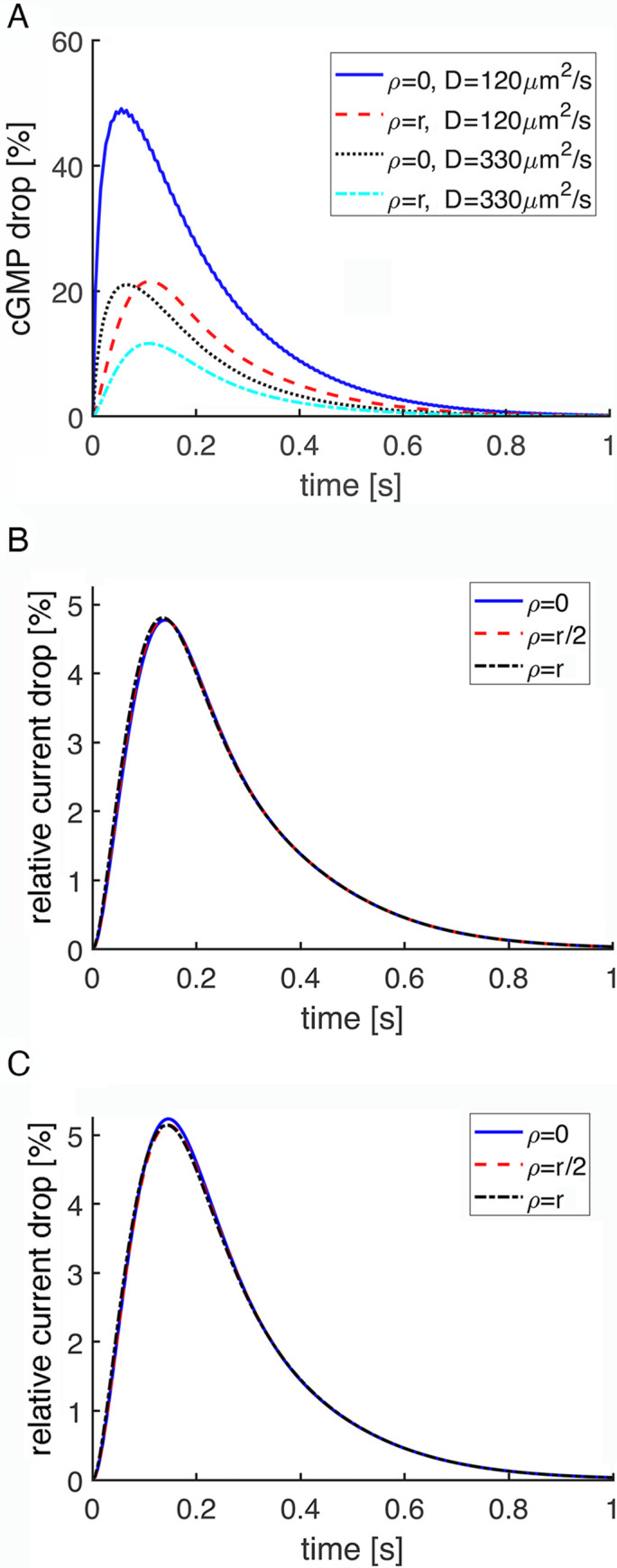
Low SPR variability due to R* position in a mouse rod despite radial gradients of cGMP. (**A**) Gradients were computed for an R* in the center of the disk for disks lacking an incisure using two different values for the diffusion coefficient of cGMP. Disk radius, r, was 0.685 μm. (**B**) Variability in SPR due to R* location was low for disks lacking the incisure. R* was placed at the disk center (ρ = 0), halfway to the rim (ρ = r/2), or at the rim (ρ = r). (**C**) The SPR was larger and variability increased, when each disk had a single incisure. The incisure penetrated radially 0.31 μm into the disk interior. Incisures were aligned in disks throughout the outer segment. D_cG_ was 120 μm^2^ s^-1^ for simulations in (**B**) and (**C**).

It can be expected that SPR variability due to randomness in the position of photoisomerization should disappear with decreasing disk size. Mouse rod disks have a radius that is ~1/10th that of the largest salamander rod disks. Nevertheless, a photoisomerization at the center of the disk produced a radial gradient in cGMP within the interdiskal space (**Fig 6A**). Inhomogeneities in cGMP levels would diminish and dissipate faster if cGMP were to diffuse very rapidly. Ranges of values have been reported for the axial diffusion of cGMP and for the tortuosity factor in the rod [36–39], so gradients were calculated for two diffusion coefficient values for cGMP that differed by 2.75-fold. The local drop in cGMP was greater with the lower diffusion coefficient, but clear gradients were formed in both cases.

As expected, rhodopsin activation position had a much smaller effect on SPR variability in mouse because all locations on the disk were proximal to the CNG channels in the plasma membrane. In deterministic runs, the SPR changed very little with R* location in the absence of the incisure (**Fig 6B**). The incisure increased SPR amplitudes somewhat (**Fig 6C**), by facilitating the axial diffusion of cGMP within the outer segment after hydrolysis at the activated disk, consistent with previous findings [24], but made the disparity between SPRs arising from photoisomerizations at the different disk locations slightly greater.

In contrast to the salamander rod SPR, a damped oscillation was not present late in the recovery of the mouse rod SPR either with or without the incisure and irrespective of R* location (**Fig 6B and 6C**). Even though the drop in cGMP was greater in mouse rods compared to that in salamander rods, the timing of events was different. In salamander rods, the return of guanylate cyclase activity towards its basal level in darkness was met with a residual PDE* activity that effected a small, secondary drop in circulating current. But in mouse rods, PDE* inactivation was already complete by the time guanylate cyclase activity recovered to its basal level in darkness, so the damped oscillation did not occur.

**Fig 7:**
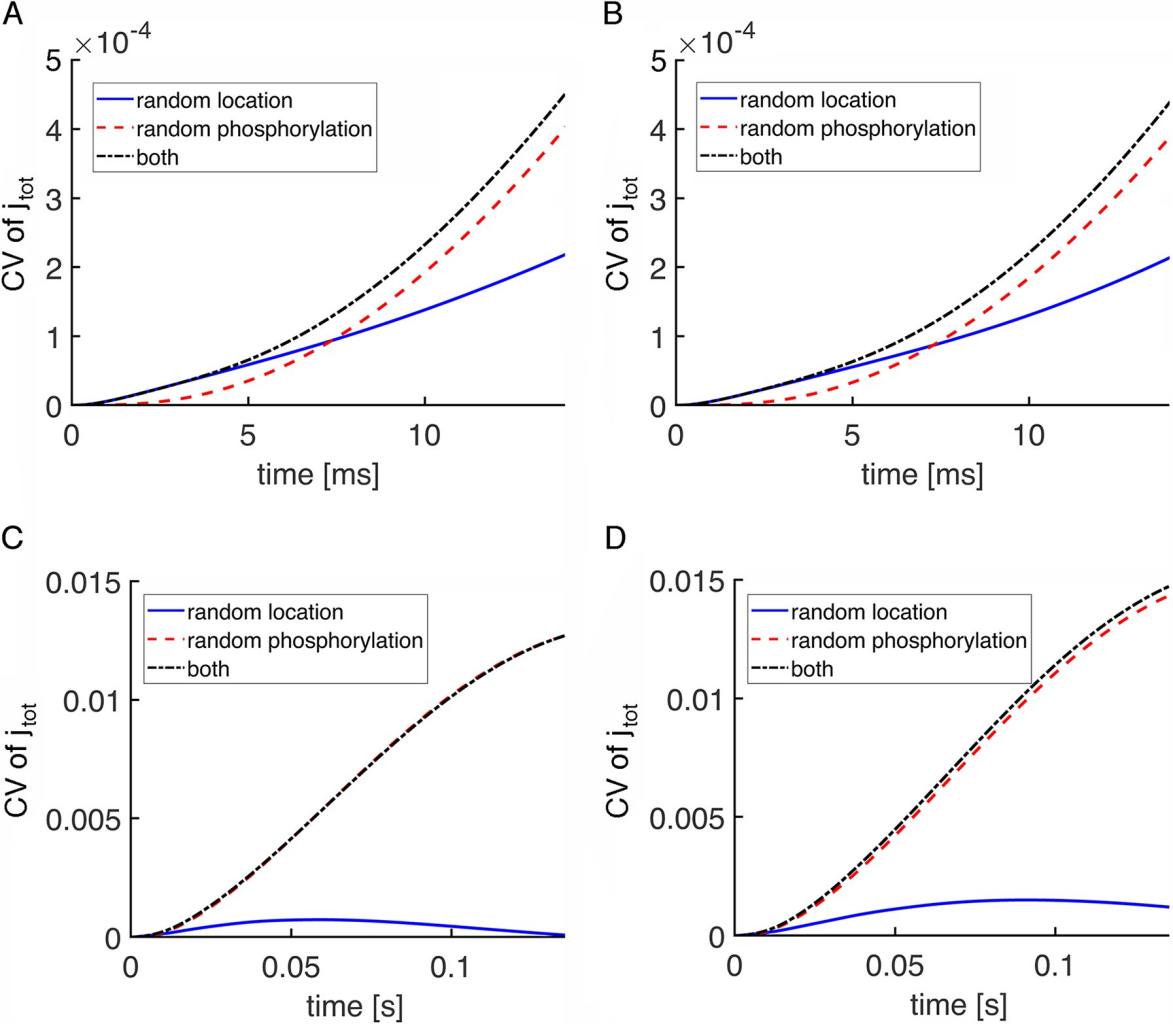
CV of the total current due to two sources of variability in a mouse SPR. Randomness in the shutoff of R* dominated CV of the total current, j_tot_(t), over most of the duration of the SPR. The exception was at very early times after photon absorption, when randomness in the location of the photoisomerization contributed more variability as shown on an expanded time scale (**A, B**). The incisure was absent in simulations in (**A, C**) and was present for simulations in (**B, D**). D_cG_ was 120 μm^2^ s^-1^.

Stochastic SPR simulations indicated that CV for j_tot_ in mouse was dominated by randomness in R* phosphorylation except for a very early segment of the rising phase (**Fig 7**). Mouse rod SPRs were experimentally shown to have faster kinetics than those of salamander rods [40, 41], as reflected in **S1** and **S2 Figs** in **S1 Appendix**, and in mouse rod simulations with stochastic R* shutoff, the average time taken for the first phosphorylation was 16 ms [11]. There were three main differences from salamander with respect to variability. First, the switch to the randomness of R* phosphorylation prevailing as the main contributor of variability occurred at ~7 ms in mouse (**Fig 7A and 7B**), much sooner than at 800 ms in salamander rods lacking incisures (**Fig 4C**) and at 220 ms in salamander rods with incisures (**Fig 5C**), due to the faster equilibration of cGMP within the smaller cytosolic volume between disks and faster phosphorylation of R* in mouse. Second, randomness in R* location contributed a negligible amount of variability in mouse even during the rising phase. Third, the presence of a single incisure in each mouse disk had the opposite effect on CV (**Fig 6B and 6C**) than the presence of multiple, symmetrically placed incisures in each salamander disk (**Figs 4 and 5**). In mouse, CV due to randomness in R* location was quite a bit higher with the incisure because it introduced spatial asymmetry to the disk surface, i.e., a diffusion barrier for R*, activated transducin, and PDE* (**Fig 7C and 7D**). The high CV of j_tot_ from both sources at the peak of the SPR in mouse rods, compared to that in salamander rods, has a trivial origin, arising from the smaller circulating current in mouse. As will be shown below, that difference is minimal in more informative comparisons of CV of the relative current drop (**Fig 10**).

### Dependence of variability on disk radius

Differences in the contribution of photoisomerization position to SPR variability between small mouse rods and large salamander rods were consistent with a dependence upon disk size. But the time course of the SPR is considerably faster in mouse than in salamander and many key phototransduction parameters vary between mouse and salamander, so the effect of geometry alone was explored by varying disk size in an idealized rod that had the physical and kinetic parameters of a salamander rod except for the removal of all incisures. Disk radius was systematically reduced from 6 μm to 1 μm, keeping constant the ionic channel density on the outer shell among the various cases. Stochastic simulations were computed by letting in each trial, the photoisomerization location be randomly chosen on the midstack disk. For reference, rod outer segment radius is 0.7 μm in mouse [5], 2.5–3 μm in toad [42] and up to 6.5 μm in salamander [6–8].

**Fig 8:**
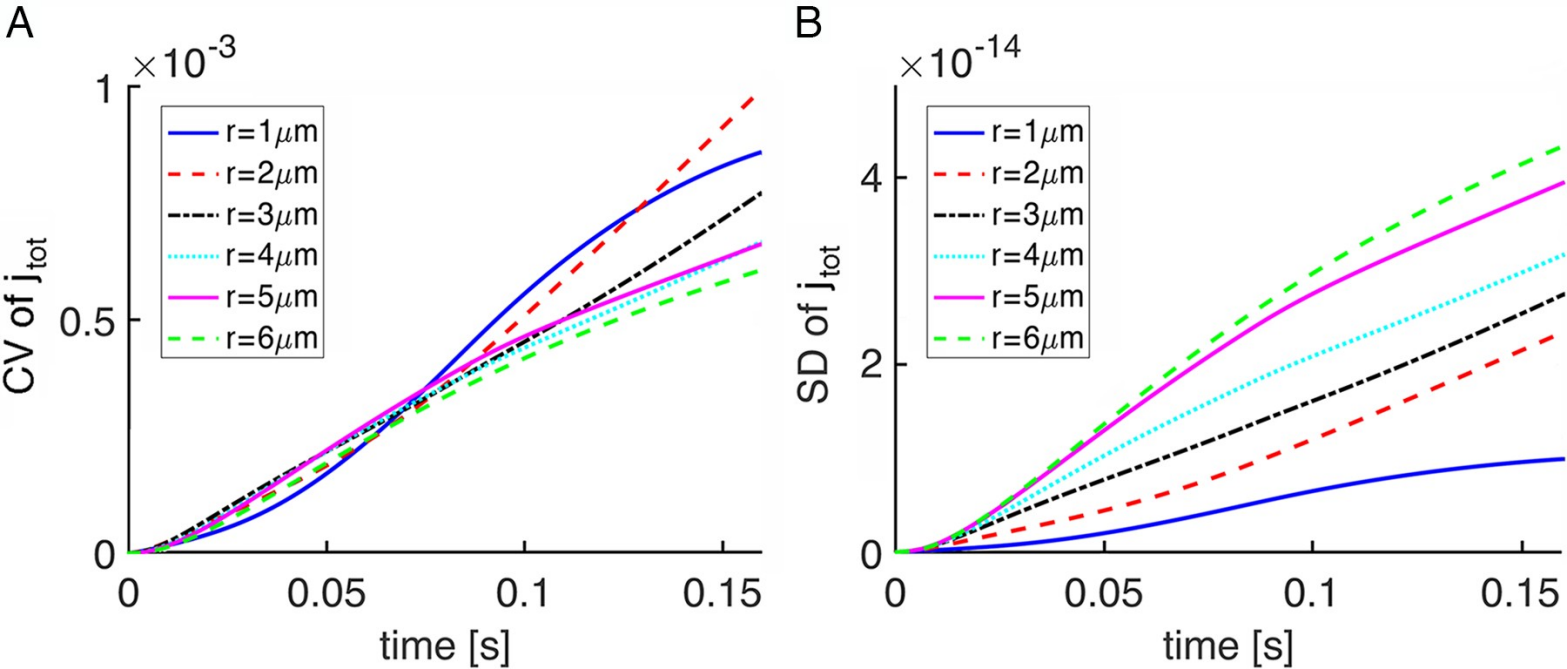
Variability in SPR total current due to R* location in salamander rods of decreasing size. (**A**) Early SPR variability due to randomness in R* location changed very little with decreasing disk radii, when assessed by CV of the total current, j_tot_(t). (**B**) In contrast, the standard deviation of the total current decreased systematically with a reduction in disk radius. Disks lacked incisures.

**Fig 9:**
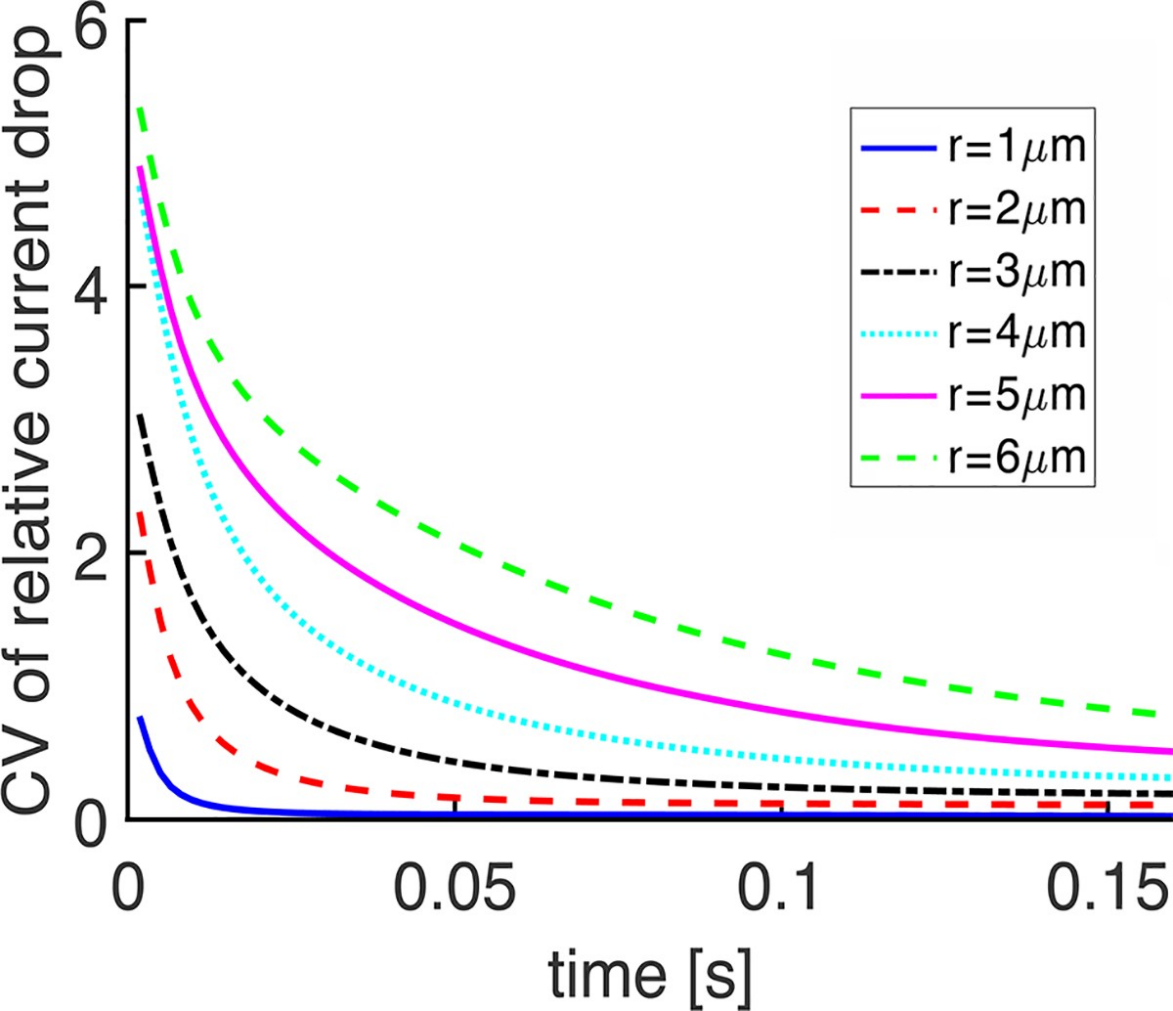
Systematic decrease in CV of I(t) with reduction in disk radius in salamander rods. R* location on the disk was chosen randomly in each trial and R* and PDE* activities declined exponentially over time. Disks lacked incisures.

Variability did not decrease systematically with diminution of radius, based on calculations of the CV of the total current (**Fig 8A**). The reason is that the results were confounded by having held constant the ionic channel density in the outer shell for all disk radii, which resulted in a decrease in circulating current with smaller radii. In computing the CV of the total current, the mean current appearing in the denominator was very large, essentially equal to the dark current, so the relatively small decreases in the standard deviation of the change in total current in large rods were offset by corresponding decreases in the circulating current. This interpretation was confirmed with plots of the standard deviation of the total current, which showed that the total current was clearly more variable in cases of larger radii (**Fig 8B**). It was therefore more instructive to express variability in terms of CV of the relative current drop, I(t) (**Fig 9**), in order to reveal the systematic decrease in variability with reduction in disk radius. During the first few ms, CV was very high for rods of all sizes due to the very small amplitude of the SPR. CV would not be biologically meaningful during this time because the response would not yet have risen out of the continuous noise of the phototransduction cascade. With small rods, CV fell to low levels rather quickly, but several hundred ms were needed for that to happen in the largest rods.

**Fig 10:**
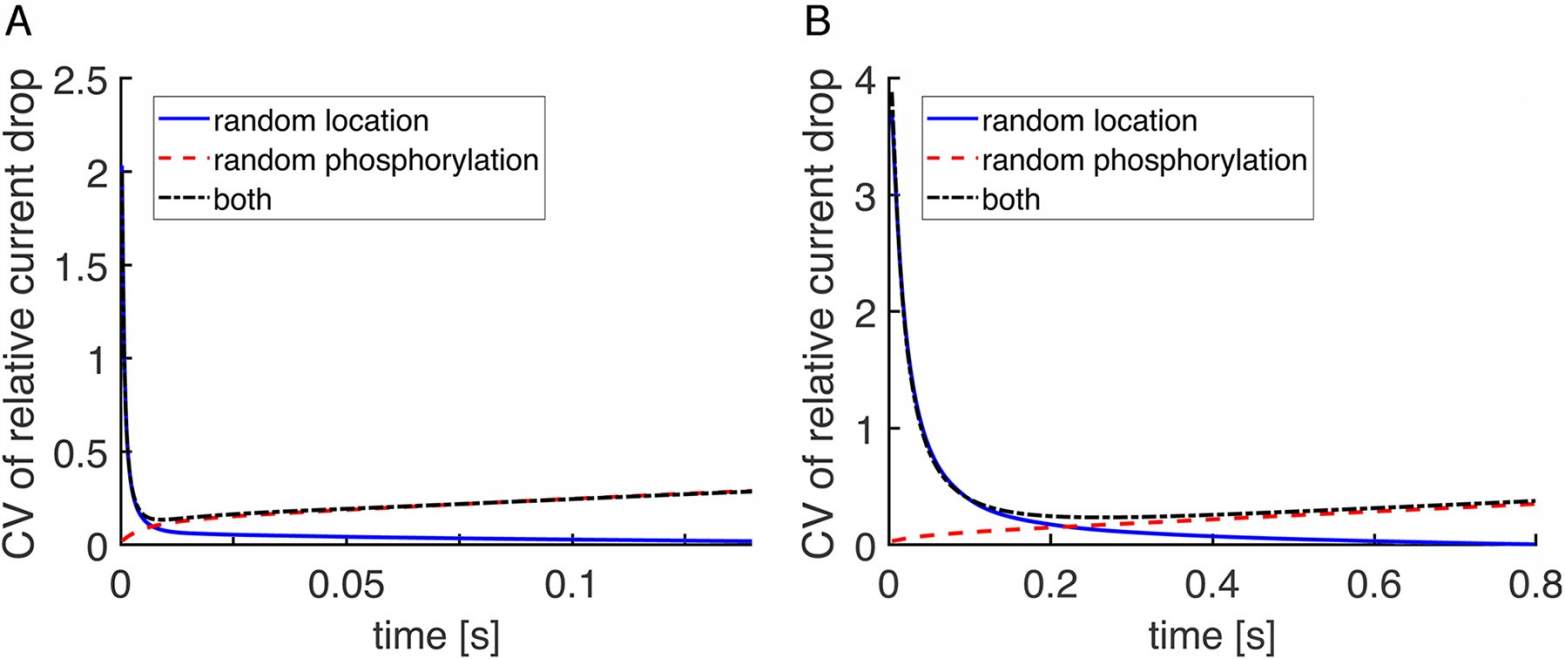
Lower initial CV of I(t) and faster decline in wild type mouse and salamander rods. CV of the relative drop in current, I(t), is plotted over time just beyond the SPR time to peak in: (**A**) a mouse rod with disk radius = 0.685 μm and one incisure and (**B**) a salamander rod with disk radius = 5.5 μm and 23 incisures.

Finally, CV of the relative drop in current, I(t), is reported, comparing results for wild type mouse (**Fig 10A**) and large, wild type salamander (**Fig 10B**) rods with incisures. The behavior of the curves led to similar conclusions for the role of the randomness in the photoisomerization site and for the role of the incisures. Notice that the curves of CV due to randomness in the activation site began with a peak. This was because the amplitude of the relative drop in current at early times was very small, yielding large CV values even for a modest standard deviation. The CV generated by both factors was higher in salamander than in mouse, consistent with results of [19], but in apparent contradiction to the results shown for the total current CV (**Figs 5** and **7**). As a matter of fact, this was only due to the mean of the total current during the SPR being close to the dark current value, which was larger in salamander than in mouse. When the relative drop in current was analyzed, the dark current was normalized, so it did not influence the CV.

In mouse, CV for the relative current drop due to randomness in the location of photoisomerization fell to ~0.09 after 7 ms, when randomness in R* phosphorylation became the dominant source of variability. In salamander, CV due to random location of photoisomerization was ~0.4 after 100 ms and was still ~0.2 after 200 ms. Thereafter, randomness in R* phosphorylation became the dominant source of variability. Hence variability due to randomness in photoisomerization location was insignificant at the peak of the salamander SPR, where CV due to randomness in R* phosphorylation was about 0.35. Experimental determinations of CV are not yet available for salamander rods, but for toad rods, that tend to have a disk radius of ~3 μm, CV at the peak of the SPR due to all sources is ~0.2 [27] and for mouse rods it is ~0.3 [43].

In conclusion, these simulations indicated that the rising phase of the SPR generated by a salamander rod with a large cross section was more prone to variability due to randomness in the location of rhodopsin photoisomerization than rods with a small cross section, such as those of mouse, which closely resemble human rods. Incisures mitigated this source of variability by reducing the magnitude and shortening the time course over which it exerted its effect in salamander. Interestingly, the single incisure in mouse disks had the opposite effect on variability due to photoisomerization location because it created a non-symmetric condition, but the overall variability remained too small to impact signaling.

## Discussion

The capacity for rhodopsin to form dimers and higher order oligomers raises questions about its mobility in the disk membrane. The lateral diffusion of rhodopsin has been measured many times in amphibian rods in the last 40 years. Early measurements of unmodified rhodopsin [26, 44], as well as rhodopsin labeled with rhodamine [45], yielded coefficients of 3–5 x 10^−9^ cm^2^ s^-1^. Rapid rotational diffusion was also consistent with a single rhodopsin molecule moving within the disk membrane. Later it became accepted that early measurements, at least those that used unmodified rhodopsin, were partially compromised by the emergence of metarhodopsin III so these measurements were performed again and yielded a more complex picture [46]. The lipid environment of disk membranes in Xenopus was shown to be non-homogeneous, with apparent lipid rafts, which could be destroyed by using cholesterol-depleting drugs [47]. In the dark and immediately after photoexcitation both rhodopsin-EGFP and EGFP-tagged transducin diffuse freely in the bulk of fluid lipid. Slowing of diffusion was observed upon the formation of the complex of R* with transducin [47]. Cholesterol depletion prevented this slowing, suggesting that these complexes either move to less fluid microdomains, or induce the formation of cholesterol-containing membrane microdomains around them. The more recent measurement was performed with unmodified rhodopsin in amphibian (frog, toad, and salamander) and gecko rods [46]. In all cases, after elimination of the metarhodopsin III contribution, rhodopsin diffusion was found to be essentially the same as previously reported, with a diffusion coefficient of 5×10^−9^ cm^2^ s^−1^. Yet the authors found that a fraction of the rhodopsin, which varied from virtually zero to 100%, was immobile. The authors were unable to establish the factor(s) that determined the size of this fraction. This immobile fraction might represent paracrystalline rhodopsin structures reported in disks immobilized on mica [48]. Govardovskii et al. [46] hypothesized that the fraction of immobile rhodopsin might be controlled physiologically to regulate the sensitivity of the phototransduction cascade, implying that only freely mobile rhodopsin mediates signaling. This is consistent with the demonstration that monomeric rhodopsin is necessary and sufficient to activate transducin [49, 50], become phosphorylated by GRKs [51, 52] and bind arrestin [51–53]. It was also established that arrestin binds rhodopsin at 1:1 ratio, both in vitro [51, 54] and in mouse photoreceptors [55]. The functional findings were further validated by the elucidation of structures of rhodopsin complexes with G protein [56], GRKs [57], and arrestin [58, 59]. At the moment, there is no evidence for the participation of anything greater than a single molecule of light-activated rhodopsin in visual signaling. Indeed, it was established long ago that rods respond to single photons [27], which can activate only one molecule of rhodopsin. Based on available information, we modeled visual signal transduction assuming free diffusion of monomeric rhodopsin within the disk membrane. In the future, it would be interesting to explore with the model, how rhodopsin dimers and higher order oligomers of immobile multi-rhodopsin formations impact cascade dynamics and the spatial distribution of cGMP over the duration of the SPR.

By assuming that radial cGMP gradients in the interdiskal space equilibrate with infinite rapidity relative to the time course of the SPR, globally well stirred and transversely well stirred models of visual transduction disregard any possibility that the transverse position of rhodopsin photoisomerization on the disk could produce variability in the SPR. The fully space-resolved model invalidated that assumption in hypothetical salamander rods lacking incisures by showing that transverse gradients of cGMP concentration were created by a photoisomerization [18, 20], that the gradients evolved over a time scale of seconds, and that the form of the gradient depended upon the radial location of the photoisomerization on the disk (**Fig 2**). Salamander disks are large, with a radius of 5.5 μm, but mouse rod disks have a radius that is 8-fold smaller, enabling cGMP gradients in the interdiskal space to dissipate more rapidly. Nevertheless, marked radial gradients in cGMP were still present late into the time course of the SPR of hypothetical mouse rods lacking incisures (**Fig 6A**). Increasing the diffusion of cGMP by nearly 3-fold reduced the magnitude of the drop in cGMP, but did little towards changing the difference in the drop in cGMP levels in the center of the disk relative to that at the rim, for a centrally located photoisomerization.

Our modeling allowed subunits of the PDE dimer to be activated individually by single GTP-bound transducin molecules (see **Methods** and **S1 Appendix**). However, a recent study found that in the presence of membrane, i.e., under native conditions, a single transducin bound to the PDE dimer produced little if any increase in hydrolytic activity [25]. PDE activation required the binding of two transducins. To test whether the requirement for two transducins to activate PDE would change our conclusions, some simulations were performed by incorporating this feature into our model. The results are shown in **S1 File**. The radial cGMP gradients produced by an R* located in the center of a mouse disk were not substantially altered (**S4 Fig**) nor were there any significant effects on CV during the rising phase of the SPR (**S5 Fig**).

Although the radial cGMP gradients gave rise to fairly minor differences in SPR kinetics and amplitudes in mouse rods (**Fig 6B and 6C**), the differences were sizeable in salamander rods (**Fig 4A**; see also **Fig 7B** in [18]). The basis for the disparity across species was explored by systematically varying disk radius (**Figs 8 and 9**). Larger disk size generated increasing variability in the rising phase of the SPR. The reason is that due to the finite diffusion rate of cGMP, longer time periods would pass between the initial, local depletion of cGMP near the photoactivation site on the disk and the reduction of cGMP at the plasma membrane, where it would lead to closure of the CNG channels. These results confirm the hypothesis that the initially high CV followed by a decline during the early rising phase of the SPR was due to randomness in the site of photoisomerization on the disk [19].

Our approach may have overestimated CV due to phosphorylation at early times because in our simulations, phosphorylation of R* began to decrease its activity within a few tens of ms after photoisomerization, whereas experimental evidence indicates that it occurs just before the peak of the SPR in mouse rods [16, 17]. The small contribution of the random rate of transducin/PDE activation to overall variability was omitted because that part of the cascade was deterministic in our simulations.

In our treatment, basal PDE activity was taken to be uniform over time and space, but in reality, it arises from spontaneous activations of PDE molecules that occur throughout the rod and generate "continuous noise" [60]. The inhomogeneity in cGMP levels over time and space caused by random PDE activations adds further variability to the early, rising phase of the SPR. It was removed in our analysis in order to isolate the contribution of photoisomerization location to variability. However, as the response to R* grows, the impact of both sources of variability diminishes as more PDE*s are recruited across the disk surface and the depletion of cGMP spreads over a greater volume.

In small diameter mouse rods, the presence of a single incisure increased the size of the SPR and enhanced its variability slightly, by allowing cGMP gradients to dissipate axially and by distorting the spread of transducin/PDE activation across the disk surface (**Fig 6B and 6C**). However, variability due to R* location was never very large in mouse rods even in the presence of an incisure and it faded after the first 100 ms (**Fig 7**). Thus, it appears that the biological role of a single incisure in relatively small disks, such as those in mouse, is not to improve SPR reproducibility.

In contrast, SPR variability was reduced by multiple incisures arranged symmetrically around the disk perimeter in salamander rods (**Figs 4** vs **5**). Nevertheless, CV of the relative current drop was still quite high in salamander rods for the first hundred ms (**Fig 10B**), consistent with [19]. The incisures acted in two ways. By cordoning off wedge shaped sections of the disk, they caused R* to activate a more uniform compartment of the disk membrane surface [19]. In addition, due to faster axial diffusion, they reduced the drop in cGMP concentration near the activated disk and spread the change over a greater length of the outer segment. The conclusion that SPRs arising from photoisomerizations in the center of the disk and at the rim would be similar in large amphibian rods with incisures as well as in small mouse rods is supported by electrophysiological recordings of single toad rods [28]. Stimulation of the rod with a narrow slit of light passing through the full diameter of its outer segment gave rise to dim flash responses that were similar in form to those with the slit placed on the edge of the outer segment. Variability for the SPRs elicited at the two slit positions was not analyzed, so the predicted difference in CV in the initial segment of the rising phase awaits confirmation. Another experimental prediction is that CV in the early rising phase of the SPR would be greater in large amphibian rods lacking incisures. This prediction is more difficult to test because thus far, rods with large disks lacking incisures have not been found in nature. Interestingly, disks in human rods are only slightly larger than those of mouse, yet form a more symmetric pattern of multiple, shallow incisures [61].

The slow time course of the rod response, which is necessary for amplification, impedes temporal resolution. To improve upon signaling of photon arrival while suppressing the high frequency continuous noise generated by the phototransduction cascade in the rod, rod signals undergo band pass filtering at the bipolar cell [4, 62]. Thus, bipolar cells "pay particular attention" to the rising phase of the SPR, which makes the variability during this period more important biologically. Variability in the rising phase of the SPR subsides quickly in small diameter mouse rods, but is more substantial and protracted in large salamander rods. A less "reliable" rising phase may explain why the rod bipolar cell response to the SPR of the rod appears to be accelerated less in salamander than in mouse [3, 4, 63]. This variability must impose a more severe limitation on the speed of salamander rod vision based on the SPRs of single rods.

Rod bipolar cells receive convergent input from 10–25 rods in salamander and in mouse [64–66]. Considering a greater than 4-fold amplification of the rod signal in the rod bipolar cell [3, 67–69], summation of the phototransduction cascade noise from all of these rods would make single photon detection impossible. As a countermeasure, rod bipolar cells perform a thresholding operation [69–71]. Release of glutamate neurotransmitter at the rod to rod bipolar cell synapse is saturating, rendering the small reductions in release due to continuous noise insufficient to elicit a postsynaptic response. Thresholding also causes the rod bipolar cell to ignore the very initial segment of the rising phase of the rod SPR, the period of greatest variability due to randomness in R* location. Thus, in addition to intra-rod mechanisms, e.g., [22], thresholding by bipolar cells contributes to the accuracy of photon counting by the visual system in dim light.

## Supporting information

S1 Appendix Fully space-resolved model and parameter sets. (PDF)

S1 File Simulations in which two transducins are necessary for PDE activation. (PDF)
