# Supplementary material for: Position of rhodopsin photoisomerization on the disk surface confers variability to the rising phase of the single photon response in vertebrate rod photoreceptors
Source: PLoS One. 2020 Oct 14;15(10):e0240527. doi: 10.1371/journal.pone.0240527 (PMC7556485; doi:10.1371/journal.pone.0240527)
Supplement: S1 Appendix — (PDF) [file pone.0240527.s001.pdf]

S1 Appendix. Fully space resolved model and parameter sets.

Position of rhodopsin photoisomerization on the disk surface confers variability to the rising phase of the single photon response in vertebrate rod photoreceptors

Giovanni Caruso
Italian National Research Council, Istituto di Scienze del Patrimonio Culturale
Via Salaria Km. 29,300- C.P. 10 Monterotondo St., Roma, Italy
email: giovanni.caruso@itabc.cnr.it

Colin J. Klaus
The Mathematical Biosciences Institute, Ohio State University,
1735 Neil Avenue, Columbus, OH 43210
email: klaus.68@mbi.osu.edu

Heidi E. Hamm
Department of Pharmacology, Vanderbilt University Medical Center,
2200 Pierce Avenue, Preston Research Building Rm 452
Nashville, TN 37232
email: heidi.hamm@vanderbilt.edu

Vsevolod V. Gurevich
Department of Pharmacology, Vanderbilt University Medical Center,
2200 Pierce Avenue, Preston Research Building Rm 452
Nashville, TN 37232
email: Vsevolod.Gurevich@vanderbilt.edu

Clint L. Makino
Department of Physiology and Biophysics, Boston University School of Medicine
W402 700 Albany Street, Boston, MA 02118-2526
email: cmakino@bu.edu

Emmanuele DiBenedetto
Department of Mathematics, Vanderbilt University
1326 Stevenson Center, Nashville, TN 37240
email: em.diben@vanderbilt.edu

A. Dynamics of the cascade

A.1 Symbolism

H = height of the ROS

r = disk radius (disregarding incisures)

D_R = disk of radius r centered at the origin of r^2

n_{inc} = number of incisures

V_j = limiting j^{th} incisures, assimilated to segments of length r $- r_{o,j}$

r_j = radial variable on V_j with origin at $r_{o,j}$

$\theta_{\varepsilon_o,j}(r_j)$ = geometry of the jth incisure with tip at $r_{o,j}$

$D_{eff} = D_R - \bigcup^{m}_{j=1} V_{j=1}$ effective domain of the activation cascade

k = number of distinct activated disks, each disk being activated by a single photon

$D^*_{i,\mathrm{eff}}$ = i^{th} activated disk

$\Omega = D_R \times (0, H)$ limiting cylinder enclosing the stack of disks D_R

$\Omega_{\mathrm{eff}} = D_{\mathrm{eff}} \times (0, H)$ limiting domain available for diffusion of cGMP and Ca^{2+}

$\mathcal{B}_j = V_j \times (0, H)$ limiting vertical rectangles cut on the limiting ROS by the limiting

incisures aligned in series

S = limiting outer shell (same as lateral boundary of Ω)

dS = surface measure on S

ε_o = disk width

$\nu\varepsilon_o$ = width of each interdiskal space

$\sigma\varepsilon_o$ = width of the outer shell

$1 - \mu_o$ = volume ratio of cytosol to the volume of the ROS

[cGMP] = cGMP concentration in the interior of the limiting ROS

$[\mathrm{cGMP}]_*$ = [cGMP] at the activated disk

$[\mathrm{cGMP}]_S$ = [cGMP] in the limiting outer shell

$[\mathrm{cGMP}]_{\mathcal{B}_j}$ = [cGMP] on $\mathcal{B}_j$

$[Ca^{2+}]$ = $[Ca^{2+}]$ in the interior of the limiting ROS

$[Ca^{2+}]_*$ = $[Ca^{2+}]$ at the activated disk

$[Ca^{2+}]_S$ = $[Ca^{2+}]$ in the limiting outer shell

$[Ca^{2+}]_{\mathcal{B}_j}$ = $[Ca^{2+}]$ on $\mathcal{B}_j$

∇_S = gradient along the cylindrical variables of S

$\nabla_{\mathcal{B}_j}$ = gradient along the (r_j, z) variables of $\mathcal{B}_j$

$\nabla_{(x,y)}$ = gradient along the horizontal variables (x, y)

A2. Weak formulation of the cGMP dynamics

$$
\begin{aligned}
(1-\mu_0)\Bigg\{ & \iiint_{\Omega_{\text{eff}}} [\text{cGMP}]\,(t)\varphi(t)dxdydz - \iiint_{\Omega_{\text{eff}}} [\text{cGMP}]_{\text{dark}}\,\varphi(0)dxdydz \\
& + \int_0^t \iiint_{\Omega_{\text{eff}}} \Big\{-[\text{cGMP}]\varphi_t + \text{D}_{\text{cG}}\nabla_{(x,y)}[\text{cGMP}]\cdot\nabla_{(x,y)}\varphi \\
& - \Big[\alpha([\text{Ca}^{2+}]) - \beta_{\text{dark}}[\text{cGMP}]\Big]\varphi\Big\}\, dxdydz\, d\tau \Bigg\}_{\text{interior}} \\
& + \nu\varepsilon_0 \Bigg\{ \sum_{i=1}^{k} \iint_{D^*_{i,\text{eff}}} \{[\text{cGMP}]_*(t)\varphi(t) - [\text{cGMP}]_{\text{dark}}\varphi(0)\}\, dxdy \\
& + \sum_{i=1}^{k} \int_0^t \iint_{D^*_{i,\text{eff}}} \Big\{-[\text{cGMP}]_*\varphi_t + \text{D}_{\text{cG}}\nabla_{(x,y)}[\text{cGMP}]_*\cdot\nabla_{(x,y)}\varphi \\
& - \left(\alpha([\text{Ca}^{2+}]_*) - \beta_{\text{dark}}[\text{cGMP}]_* - \frac{\text{k}^*_{\sigma;\text{hyd}}}{\nu\varepsilon_0}[\text{E}^*]_\sigma[\text{cGMP}]_*\right)\varphi\Big\}\, dxdyd\tau \Bigg\}_{\text{activated discs}} \\
& + \sigma\varepsilon_0 \Bigg\{ \iint_S \{[\text{cGMP}]_S(t)\varphi(t) - [\text{cGMP}]_{\text{dark}}\varphi(0)\}\, dS \\
& + \int_0^t \iint_S \{-[\text{cGMP}]_S\varphi_t + \text{D}_{\text{cG}}\nabla_S[\text{cGMP}]_S\cdot\nabla_S\varphi\} dSd\tau \Bigg\}_{\text{outer shell}} \\
& + 2\Bigg\{ \sum_{j=1}^{m} \iint_{\mathcal{B}_j} r_j\theta_{j,\varepsilon_0}(r_j)\Big\{[\text{cGMP}]_{\mathcal{B}_j}(t)\varphi(t)dr_jdz - [\text{cGMP}]_{\text{dark}}\varphi(0)\Big\}\, dr_jdz \\
& + \sum_{j=1}^{m} \int_0^t \iint_{\mathcal{B}_j} r_j\theta_{j,\varepsilon_0}(r_j)\Big\{[\text{cGMP}]_{\mathcal{B}_j}\varphi_t + \text{D}_{\text{cG}}\nabla_{\mathcal{B}_j}[\text{cGMP}]_{\mathcal{B}_j}\cdot\nabla_{\mathcal{B}_j}\varphi\Big\}\, dr_jdzd\tau \Bigg\}_{\text{incisures}} = 0
\end{aligned}
$$

for all $t > 0$ and all smooth, real valued functions φ in $\overline{\Omega} \times \mathbb{R}^+$. Here

$$\alpha([\text{Ca}^{2+}]) = \alpha_{\min} + (\alpha_{\max} - \alpha_{\min})\frac{\text{K}_{\text{cyc}}^{\text{m}_{\text{cyc}}}}{\text{K}_{\text{cyc}}^{\text{m}_{\text{cyc}}} + [\text{Ca}^{2+}]^{\text{m}_{\text{cyc}}}}$$

$$\alpha([\text{Ca}^{2+}]_*) = \alpha_{\min} + (\alpha_{\max} - \alpha_{\min})\frac{\text{K}_{\text{cyc}}^{\text{m}_{\text{cyc}}}}{\text{K}_{\text{cyc}}^{\text{m}_{\text{cyc}}} + [\text{Ca}^{2+}]_*^{\text{m}_{\text{cyc}}}}$$

where $\alpha_{\max} = \text{k}_{\text{GC, max}}[\text{GC}]$ and $\alpha_{\min} = \text{k}_{\text{GC, min}}[\text{GC}]$ and $\text{k}_{\text{GC, max}}$ and $\text{k}_{\text{GC, min}}$ are the maximal and minimal catalytic rates of cGMP synthesis by guanylate cyclase GC occurring as $[\text{Ca}^{2+}] \to 0$ and as $[\text{Ca}^{2+}] \to \infty$, respectively.

A3. Weak formulation of the Ca^{2+}dynamics

$$
\begin{aligned}
(\mathbf{1}-\boldsymbol{\mu}_0)\Bigg\{&\iiint_{\Omega_{\text{eff}}}[\text{Ca}^{2+}]\,(t)\varphi(t)\,dxdydz-[\text{Ca}^{2+}]_{\text{dark}}\varphi(0)\,dxdydz\\
&+\int_0^t\iiint_{\Omega_{\text{eff}}}\{-[\text{Ca}^{2+}]\varphi_t+\text{D}_{\text{Ca}}\nabla_{(x,y)}[\text{Ca}^{2+}]\cdot\nabla_{(x,y)}\varphi\}\,dxdydz\,d\tau\Bigg\}_{\text{interior}}\\
&+\nu\varepsilon_0\Bigg\{\sum_{i=1}^{k}\iint_{D^*_{i,\text{eff}}}\{[\text{Ca}^{2+}]_*(t)\varphi(t)-[\text{Ca}^{2+}]_{\text{dark}}\varphi(0)\}\,dxdy\\
&+\sum_{i=1}^{k}\int_0^t\iint_{D^*_{i,\text{eff}}}\{-[\text{Ca}^{2+}]_*\varphi_t+\text{D}_{\text{Ca}}\nabla_{(x,y)}[\text{Ca}^{2+}]_*\cdot\nabla_{(x,y)}\varphi\}\,dxdy\,d\tau\Bigg\}_{\text{activated discs}}\\
&+\sigma\varepsilon_0\Bigg\{\iint_S\{[\text{Ca}^{2+}]_S(t)\varphi(t)-[\text{Ca}^{2+}]_{\text{dark}}\varphi(0)\}\,dS\\
&+\int_0^t\iint_S\{-[\text{Ca}^{2+}]_S\varphi_t+\text{D}_{\text{Ca}}\nabla_S[\text{Ca}^{2+}]_S\cdot\nabla_S\varphi\}\,dSd\tau\\
&+\int_0^t\iint_S\frac{1}{\sigma\varepsilon_0\text{B}_{\text{Ca}}\mathcal{F}}\left\{\frac{\text{j}_{\text{ex}}^{\text{sat}}}{\sum_{\text{rod}}}\frac{[\text{Ca}^{2+}]_S}{\text{K}_{\text{ex}}+[\text{Ca}^{2+}]_S}-\frac{1}{2}\text{f}_{\text{Ca}}\frac{\text{j}_{\text{cG}}^{\max}}{\sum_{\text{rod}}}\frac{[\text{cGMP}]_S^{\text{m}_{\text{cG}}}}{\text{K}_{\text{cG}}^{\text{m}_{\text{cG}}}+[\text{cGMP}]_S^{\text{m}_{\text{cG}}}}\right\}\varphi\,dSd\tau\Bigg\}_{\text{outer shell}}\\
&+2\Bigg\{\sum_{j=1}^{m}\iint_{\mathcal{B}_j}r_j\theta_{j,\varepsilon_0}(r_j)\Big\{[\text{Ca}^{2+}]_{\mathcal{B}_j}(t)\varphi(t)-[\text{Ca}^{2+}]_{\text{dark}}\varphi(0)\Big\}\,dr_jdz\\
&+\sum_{j=1}^{m}\int_0^t\iint_{\mathcal{B}_j}r_j\theta_{j,\varepsilon_0}(r_j)\Big\{[\text{Ca}^{2+}]_{\mathcal{B}_j}\varphi_t+\text{D}_{\text{Ca}}\nabla_{\mathcal{B}_j}[\text{Ca}^{2+}]_{\mathcal{B}_j}\cdot\nabla_{\mathcal{B}_j}\varphi\Big\}\,dr_jdzd\tau\Bigg\}_{\text{incisures}}=\mathbf{0}
\end{aligned}
$$

for all $t > 0$ and all smooth, real valued functions φ in $\overline{\Omega} \times \mathbb{R}^+$.

A4. Weak formulation of the dynamics of transducer and effector

$$
\iint_{D_{\text{eff}}}[\text{T}^*](t)\,\varphi(t)dxdy+\int_0^t\iint_{D_{\text{eff}}}\{-[\text{T}^*]\varphi_t+D_T\nabla[\text{T}^*]\cdot\nabla\varphi\}\,dxdyd\tau
$$

$$
=\int_0^t v_j\,\varphi(x(\tau),y(\tau)\,\tau)d\tau-\int_0^t\iint_{D_{\text{eff}}}\text{k}_{\text{T*E}}\,[\text{E}][\text{T}^*]\varphi dxdyd\tau
$$

$$
\iint_{D_{\text{eff}}}[\text{E}^*](t)\,\varphi(t)dxdy+\int_0^t\iint_{D_{\text{eff}}}\{-[\text{E}^*]\varphi_t+D_E\nabla[\text{E}^*]\cdot\nabla\varphi\}\,dxdyd\tau
$$

$$
=\int_0^t\iint_{D_{\text{eff}}}\{\text{k}_{\text{T*E}}[\text{E}][\text{T}^*]\varphi-\text{k}_{\text{E}}[\text{E}^*]\varphi\}\,dxdyd\tau
$$

for all $t > 0$ and all smooth, real valued functions φ in $\overline{D_R} \times \mathbb{R}^+$. Here ν_j is the rate of activation of T* by rhodopsin in its jth random phosphorylation state, i.e., when j – 1 phosphorylations have occurred (cf. [5] and **Tables S1-S4**). This presentation of the model is adapted from Bisegna et al., Biophysical Journal 2008; 94: 3363-3383.

B. Parameter selection

B.1 Salamander

Table S1: Parameters for salamander ROS

Symbol	Units	Definition	Value	References
α_{max}	$\mu M\ s^{-1}$	Maximal rate of cGMP synthesis at low [Ca^{2+}]	50	[48, 54]
$\alpha_{max}/\alpha_{min}$	-	Ratio of α from high to low [Ca^{2+}]	50	[48, 54]
A_{inc}	μm^2	Incisure area	0.8	[49]
β_{dark}	s^{-1}	Basal rate of cGMP hydrolysis by PDE in darkness	1	[6, 7, 48, 54]
B_{cG}	-	Buffering power for cGMP in cytoplasm	1	[48, 53, 54]
B_{Ca}	-	Buffering power for Ca^{2+} in cytoplasm	20	[47, 48, 54]
c_{TE}	-	Coupling coefficient of T^* to E^*	1	[34, 54]
$[cGMP]_{dark}$	μM	[cGMP] in darkness	3.0046	[30, 48]
$[Ca^{2+}]_{dark}$	nM	[Ca^{2+}] in darkness	653.7	[30, 59]
D_{cG}	$\mu m^2\ s^{-1}$	Diffusion coefficient of cGMP	160	[7, 26, 49]
D_{Ca}	$\mu m^2\ s^{-1}$	Diffusion coefficient of Ca^{2+}	15	[46]
D_{E^*}	$\mu m^2\ s^{-1}$	Diffusion coefficient of E*	0.8	[53]
D_{T^*}	$\mu m^2\ s^{-1}$	Diffusion coefficient of T*	1.5	[53]
D_{R^*}	$\mu m^2\ s^{-1}$	Diffusion coefficient of R*	0.7	[53]
ε_o	nm	Disk thickness	14	[30, 54]
η	nm	Volume-to-surface ratio	7	
$\mathcal{F}$	$C\ mol^{-1}$	Faraday's constant	96500	[48, 54]
f_{Ca}	-	Fraction of cGMP-activated current carried by Ca^{2+}	0.17	[48, 54]
H	μm	Height of ROS	22.4	[10, 23, 43, 54]
j_{dark}	pA	Dark current	65.862	[54]
j_{cG}^{max}	pA	Maximum CNG channel current	7000	[48]
j_{ex}^{sat}	pA	Saturated exchanger current	17	[54]
k_{cat}/K_m	$\mu M^{-1}\ s^{-1}$	Hydrolytic efficiency of light-activated PDE dimer	400	[34, 48, 53]
$k_{\sigma;hyd}$	$\mu m^3\ s^{-1}$	Surface rate of cGMP hydrolysis by dark-activated PDE	7×10^{-5}	[7]
$k^*_{\sigma;hyd}$	$\mu m^3\ s^{-1}$	Surface rate of cGMP hydrolysis by light-activated PDE	0.5	[1, 7]
k_E	s^{-1}	Rate constant for PDE* inactivation	0.58	[47]
k_R	s^{-1}	Rate constant for R* deactivation	2.5	[47]
k_{T^*E}	$\mu m^2\ s^{-1}$	Kinetic constant of T^*-E binding and thus E^*production	1	[56]
K_{cyc}	nM	Half-saturating [Ca^{2+}] for GC activity	135	[3, 48]
K_{cG}	μM	[cGMP] for half-maximal CNG channel opening	20	[30, 48, 54]
K_{ex}	μM	[Ca^{2+}] for half-maximal exchanger rate	1.5	[30, 48]
ℓ_b	μm	Incisure width	15	[49]
ℓ_r	μm	Incisure length	4.6377	[6]
ν	-	Ratio of interdiskal space to disk thickness	1	
$\nu\varepsilon_o$	nm	Interdiskal space	14	[30, 54]
ν_{RG}	s^{-1}	Rate of T* formation by R*	185	[22, 34, 48, 54]

n	-	Number of disks	800	
n_{inc}	-	Number of incisures	23	[17, 43, 50, 58, 68]
N_{Av}	# mol^{-1}	Avogadro number	6.02×10^{23}	
m_{cyc}	-	Hill coefficient for GC effect	2	[54]
m_{cG}	-	Hill coefficient for CNG channels	2.5	[54]
PDE^*	# μm^{-2}	Surface density of active PDE in darkness	100	[54]
r	μm	Disk radius	5.5	[10, 23, 43, 65]
σ	-	Ratio of outer shell thickness to disk thickness	15/14	
$\sigma\varepsilon_o$	nm	Distance separating disk rim from plasma membrane (outer shell thickness)	15	[30, 48]
Σ_{rod}	μm^2	Lateral surface area of a ROS	773.5	[30]
V_{cyt}	μm^3	Cytoplasmic volume	1076	[30, 54]

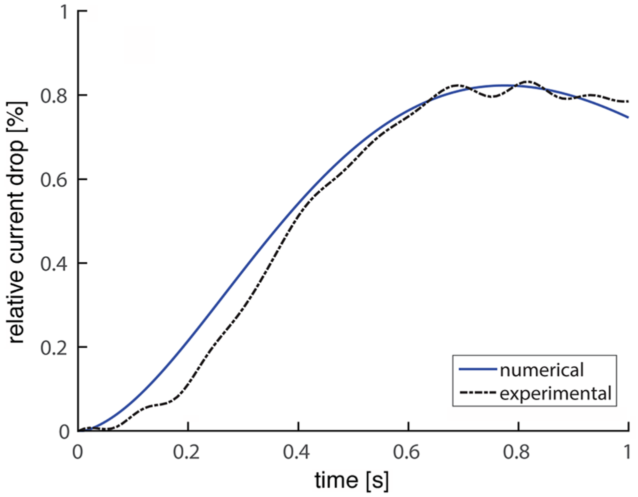

Fig S1. Simulation of the experimentally observed SPR of salamander. Experimental trace (black discontinuous line) was kindly provided by F. Rieke. Numerical simulation was performed as reported in [7] using the fully space resolved model with the parameters from **Tables S1** and **S2** (blue line).

Table S2: Deactivation parameters in the continuous time Markov Chain (CTMC) model for salamander

Symbol	Units	Definition	Value
λ_o	s^{-1}	R* phosphorylation rate	2.0
μ_o	s^{-1}	Arrestin binding rate	10
k_ν	-	Decay constant of catalytic activity of R*	0.41
$\tau_{R,eff}$	ms	Average lifetime of R*	0.4
N	-	Average number of R* phosphorylations before shut-off	4.45

These parameters were determined as described in [5]. In particular, λ_o and μ_o were chosen to ensure,

as for mouse, that the average lifetime of R* was $0.5t_{peak}$. The parameters k_ν = 0.41 and ν_{RG} = 185 s^{-1} were chosen to fit the experimental SPR curve reported in [7] and kindly provided by F. Rieke.

B2. Mouse

Table S3: **Parameters for mouse ROS**

Symbol	Units	Definition	Value	References
α_{max}	$\mu M\ s^{-1}$	Maximal rate of cGMP synthesis at low $[Ca^{2+}]$	76.5	[3, 67]
$\alpha_{max}/\alpha_{min}$	-	Ratio of α from high to low $[Ca^{2+}]$	13.9	[2, 3, 67]
A_{inc}	μm^2	Incisure area	0.0403	
β_{dark}	s^{-1}	Basal rate of cGMP hydrolysis by PDE in darkness	2.9	[8, 67]
B_{cG}	-	Buffering power for cGMP in cytoplasm	1	[53, 54]
B_{Ca}	-	Buffering power for Ca^{2+} in cytoplasm	20	[46, 47, 48]
c_{TE}	-	Coupling coefficient of T^* to E^*	1	[34, 54]
$[cGMP]_{dark}$	μM	[cGMP] in darkness	3.8	[2, 30, 48, 51, 52, 53, 54, 67]
$[Ca^{2+}]_{dark}$	nM	$[Ca^{2+}]$ in darkness	344	[16, 37, 41, 72]
D_{cG}	$\mu m^2\ s^{-1}$	Diffusion coefficient of cGMP	120	[7, 24, 49]
D_{Ca}	$\mu m^2\ s^{-1}$	Diffusion coefficient of Ca^{2+}	15	[46]
D_{E^*}	$\mu m^2\ s^{-1}$	Diffusion coefficient of E*	1.2	[53]
D_{T^*}	$\mu m^2\ s^{-1}$	Diffusion coefficient of T*	2.2	[53]
D_{R^*}	$\mu m^2\ s^{-1}$	Diffusion coefficient of R*	1.5	[53]
ε_o	nm	Disk thickness	14.5	[4, 19, 53]
η	nm	Volume-to-surface ratio	7.25	
$\mathcal{F}$	$C\ mol^{-1}$	Faraday's constant	96500	
f_{Ca}	-	Fraction of cGMP-activated current carried by Ca^{2+}	0.06	[3, 39, 54, 57, 61]
H	μm	Height of ROS	23.6	[4, 14, 15, 31, 32, 36, 38]
j_{dark}	pA	Dark current	10.9	[2, 3, 8, 9, 18, 27, 28, 44, 54, 71, 73]
j_{cG}^{max}	pA	Maximum CNG channel current	3550	
j_{ex}^{sat}	pA	Saturated exchanger current	1.8	[60, 62, 63]
k_{cat}/K_m	$\mu M^{-1}\ s^{-1}$	Hydrolytic efficiency of light-activated PDE dimer	540	[34, 53, 55]
$k_{\sigma;hyd}$	$\mu m^3\ s^{-1}$	Surface rate of cGMP hydrolysis by dark-activated PDE	2.8×10^{-5}	
$k^*_{\sigma;hyd}$	$\mu m^3\ s^{-1}$	Surface rate of cGMP hydrolysis by light-activated PDE	0.9	
k_E	s^{-1}	Rate constant for PDE* inactivation	6.5	[8, 25, 29, 39]
k_R	s^{-1}	Rate constant for R* deactivation	8.5	[8, 29, 47]

k_{T^*E}	$\mu m^2\ s^{-1}$	Kinetic constant of T^*-E binding and thus E^* production	1	[56]
K_{cyc}	nM	Half-saturating [Ca^{2+}] for GC activity	100	[3, 40, 42, 67]
K_{cG}	μM	[cGMP] for half-maximal CNG channel opening	20	[54]
K_{ex}	μM	[Ca^{2+}] for half-maximal exchanger rate	1.6	[54, 62]
ℓ_b	μm	Incisure width	0.2593	[11]
ℓ_r	μm	Incisure length	0.3111	[11]
ν	-	Ratio of interdiskal space to disk thickness	1	[4, 34, 53, 54]
$\nu\varepsilon_o$	nm	Interdiskal space	14.5	[4, 20, 36, 38, 53]
ν_{RG}	s^{-1}	Rate of T* formation by R*	330	[22]
n	-	Number of disks	814	
n_{inc}	-	Number of incisures	1	[4, 11, 53]
N_{Av}	# mol^{-1}	Avogadro number	6.02×10^{23}	
m_{cyc}	-	Hill coefficient for GC effect	2	[2, 3, 40, 42, 67]
m_{cG}	-	Hill coefficient for CNG channels	3.5	[2, 8, 45, 53, 67]
PDE^*	# μm^{-2}	Surface density of active PDE in darkness	750	[19, 36, 53, 54, 66]
r	μm	Disk radius	0.685	[4, 19, 33, 35, 36, 38]
σ	-	Ratio of outer shell thickness to disk thickness	15/14.5	
$\sigma\varepsilon_o$	nm	Distance separating disk rim from plasma membrane (outer shell thickness)	15	[12, 13, 20, 53]
Σ_{rod}	μm^2	Lateral surface area of a ROS	103.8	
V_{cyt}	μm^3	Cytoplasmic volume	18.16	

Table S4: **Deactivation parameters in the continuous time Markov Chain (CTMC) model for mouse**

**Symbol**	**Units**	**Definition**	**Value**	**References**
λ_o	s^{-1}	R* phosphorylation rate	10.5	[5]
μ_o	s^{-1}	Arrestin binding rate	60	[5]
k_ν	-	Decay constant of catalytic activity of R*	0.5	[70]
$\tau_{R,eff}$	ms	Average lifetime of R*	75	[29]
N	-	Average number of R* phosphorylations before shut-off	4.45	[5]

These parameters were determined according to [5] and calibrated to ensure that the average lifetime $\tau_{R,eff}$ of R* was $0.5t_{peak}$. The value t_{peak} obtained for $D_{cG} = 330\ \mu m^2\ s^{-1}$ was essentially the same as for $D_{cG} = 120\ \mu m^2\ s^{-1}$, so that the parameters in the table based on $\tau_{R,eff} \approx 0.5t_{peak}$ remained unchanged for these two values of D_{cG}.

C. Calibrating the mouse activation parameter ν_{RG} for D_{cG} = 330 μm^2 s^{-1}

The model parameters for mouse, including the volumic diffusivity D_{cG} = 120 μm^2 s^{-1} were chosen and justified in [5, 64], and reported here in **Tables S3** and **S4**. The diffusivity D_{cG} = 330 μm^2 s^{-1} proposed in [21], was imported here by keeping all the remaining parameters unchanged except the catalytic activity ν_{RG}, which was adjusted from 330 s^{-1} to 237 s^{-1} to reproduce the experimental SPR of [3, 5] (**Fig S2** in **S1 Appendix**).

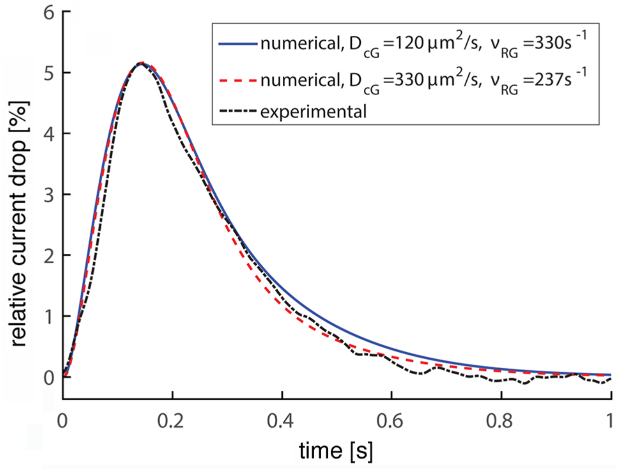

Fig S2. FSR simulations of the experimentally observed mouse rod SPR. The experimental trace (black discontinuous line) was taken from [3]. Two numerical simulations were performed: using the parameters from **Tables S3** and **S4** (red dashed line) and upon raising D_{cG} to 330 μm^2 s^{-1} with a concomitant lowering of ν_{RG} to 237 s^{-1} (blue line).

D. Relating volumic and longitudinal diffusivities

The longitudinal diffusivity D^{ℓ}_{cG} along the axis of the ROS, can be derived from the volumic D_{cG} by $D^{\ell}_{cG} = (f_A/f_V)D_{cG}$, where f_A and f_V are two geometric parameters computed in [6] as

$$f_A = \frac{A_{inc} + A_{gap}}{\pi(r + \sigma\varepsilon_0)^2 + A_{gap}}; \quad f_V = \frac{\pi r^2 + 2(A_{inc} + A_{gap})}{2(\pi(r + \sigma\varepsilon_0)^2 + A_{gap})}$$

A_{inc} is the total cross-sectional area of the incisures, A_{gap} is the cross-sectional area of the outer shell and $r + \sigma\varepsilon_0$ is the cross-sectional radius of the ROS.

References

1. Andreucci D, Bisegna P, Caruso G, Hamm HE, DiBenedetto E. Mathematical model of the spatio-temporal dynamics of second messengers in visual transduction. Biophys J. 2003; 85: 1358-1376.
2. Burns ME, Mendez A, Chen J, Baylor DA. Dynamics of cyclic GMP synthesis in retinal rods. Neuron. 2002; 36: 81-91.
3. Calvert PD, Govardovskii VI, Krasnoperova N, Anderson RE, Lem J, Makino CL. Membrane protein

diffusion sets the speed of rod phototransduction. Nature. 2001; 411: 90-94.

4. Carter-Dawson LD, LaVail MM. Rods and cones in the mouse retina. I. Structural analysis using light and electron microscopy. J Comp Neurol. 1979; 188: 245-262.
5. Caruso G, Bisegna P, Lenoci L, Andreucci D, Gurevich VV, Hamm HE, DiBenedetto E. Kinetics of rhodopsin deactivation and its role in regulating recovery and reproducibility of rod photoresponse. PLoS Comput Biol. 2010; 6: e1001031. doi:10.1371/journal.pcbi.1001031.
6. Caruso G, Bisegna P, Shen L, Andreucci D, Hamm HE, DiBenedetto E. Modeling the role of incisures in vertebrate phototransduction. Biophys J. 2006; 91: 1192-1212.
7. Caruso G, Khanal H, Alexiades V, Rieke F, Hamm HE, DiBenedetto E. Mathematical and computational modelling of spatio-temporal signalling in rod phototransduction. IEE Proc Syst Biol. 2005; 152: 119-137.
8. Chen CK, Burns ME, He W, Wensel TG, Baylor DA, Simon MI. Slowed recovery of rod photoresponse in mice lacking the GTPase accelerating protein RGS9-1. Nature. 2000; 403: 557-560.
9. Chen CK, Burns ME, Spencer M, Niemi GA, Chen J, Hurley JB, Baylor DA, Simon MI. Abnormal photoresponses and light-induced apoptosis in rods lacking rhodopsin kinase. Proc Natl Acad Sci USA. 1999; 96: 3718-3722.
10. Chen Y, Znoiko S, DeGrip WJ, Crouch RK, Ma JX. Salamander blue-sensitive cones lost during metamorphosis. Photochem Photobiol. 2008; 84: 855-862.
11. Cohen AI. The ultrastructure of the rods of the mouse retina. Am J Anat. 1960; 107: 23-48.
12. Corless JM, Fetter RD, Costello MJ. Structural features of the terminal loop region of frog retinal rod outer segment disk membranes: I. Organization of lipid components. J Comp Neurol. 1987; 257: 1-8.
13. Corless JM, Fetter RD, Zampighi OB, Costello MJ, Wall-Buford DL. Structural features of the terminal loop region of frog retinal rod outer segment disk membranes: II. Organization of the terminal loop complex. J Comp Neurol. 1987; 257: 9-23.
14. Daly GH, DiLeonardo JM, Balkema NR, Balkema GW. The relationship between ambient lighting conditions, absolute dark-adapted thresholds, and rhodopsin in black and hypopigmented mice. Vis Neurosci. 2004; 21: 925-934.
15. Daniele LL, Insinna C, Chance R, Wang J, Nikonov SS, Pugh EN Jr. A mouse M-opsin monochromat: retinal cone photoreceptors have increased M-opsin expression when S-opsin is knocked out. Vision Res. 2011; 51: 447-458.
16. Dizhoor AM, Woodruff ML, Olshevskaya EV, Cilluffo MC, Cornwall MC, Sieving PA, Fain GL. Night blindness and the mechanism of constitutive signaling of mutant G90D rhodopsin. J Neurosci. 2008; 28: 11662-11672.
17. Eckmiller MS. Microtubules in a rod-specific cytoskeleton associated with outer segment incisures. Vis Neurosci. 2000; 17: 711-722.
18. Fan J, Woodruff ML, Cilluffo MC, Crouch RK, Fain GL. Opsin activation of transduction in the rods of

dark-reared Rpe65 knockout mice. J Physiol (Lond). 2005; 568: 83-95.

19. Fotiadis D, Liang Y, Filipek S, Saperstein DA, Engel A, Palczewski K. Atomic-force microscopy: Rhodopsin dimers in native disc membranes. Nature. 2003; 421: 127-128.
20. Gilliam JC, Chang JT, Sandoval IM, Zhang Y, Li T, Pittler SJ, Chiu W, Wensel TG. Three-dimensional architecture of the rod sensory cilium and its disruption in retinal neurodegeneration. Cell. 2012; 151: 1029-1041.
21. Gross OP, Pugh EN Jr, Burns ME. Spatiotemporal cGMP dynamics in living mouse rods. Biophys J. 2012; 102: 1775-1784.
22. Hamer RD, Nicholas SC, Tranchina D, Liebman PA, Lamb TD. Multiple steps of phosphorylation of activated rhodopsin can account for the reproducibility of vertebrate rod single-photon responses. J Gen Physiol. 2003; 122: 419-444.
23. Hárosi FI. Absorption spectra and linear dichroism of some amphibian photoreceptors. J Gen Physiol. 1975; 66: 357-382.
24. Holcman D, Korenbrot JI. Longitudinal diffusion in retinal rod and cone outer segment cytoplasm: the consequence of cell structure. Biophys J. 2004; 86: 2566-2582.
25. Kennedy MJ, Sowa ME, Wensel TG, Hurley JB. Acceleration of key reactions as a strategy to elucidate the rate-limiting chemistry underlying phototransduction inactivation. Invest Ophthalmol Vis Sci. 2003; 44: 1016-1022.
26. Koutalos Y, Nakatani K, Yau KW. Cyclic GMP diffusion coefficient in rod photoreceptor outer segments. Biophys J. 1995; 68: 373-382.
27. Krispel CM, Chen CK, Simon MI, Burns ME. Novel form of adaptation in mouse retinal rods speeds recovery of phototransduction. J Gen Physiol. 2003; 122: 703-712.
28. Krispel CM, Chen CK, Simon MI, Burns ME. Prolonged photoresponses and defective adaptation in rods of Gβ5-/- mice. J Neurosci. 2003; 23: 6965-6971.
29. Krispel CM, Chen D, Melling N, Chen YJ, Martemyanov KA, Quillinan N, Arshavsky VY, Wensel TG, Chen CK, Burns ME. RGS expression rate-limits recovery of rod photoresponses. Neuron. 2006; 51: 409-416.
30. Lamb TD, Pugh EN Jr. A quantitative account of the activation steps involved in phototransduction in amphibian photoreceptors. J Physiol (Lond). 1992; 449: 719-758.
31. LaVail MM. Kinetics of rod outer segment renewal in the developing mouse retina. J Cell Biol. 1973; 58: 650-661.
32. LaVail MM, White MP, Gorrin GM, Yasumura D, Porrello KV, Mullen RJ. Retinal degeneration in the nervous mutant mouse. I. Light microscopic cytopathology and changes in the interphotoreceptor matrix. J Comp Neurol. 1993; 333: 168-181.
33. Lem J, Krasnoperova NV, Calvert PD, Kosaras B, Cameron DA, Nicolò M, Makino CL, Sidman RL. Morphological, physiological, and biochemical changes in rhodopsin knockout mice. Proc Natl Acad Sci USA. 1999; 96: 736-741.

34. Leskov IB, Klenchin VA, Handy JW, Whitlock GG, Govardovskii VI, Bownds MD, Lamb TD, Pugh EN Jr, Arshavsky VY. The gain of rod phototransduction: reconciliation of biochemical and electrophysiological measurements. Neuron. 2000; 27: 525-537.
35. Liang Y, Fotiadis D, Filipek S, Saperstein DA, Palczewski K, Engel A. Organization of the G protein-coupled receptors rhodopsin and opsin in native membranes. J Biol Chem. 2003; 278: 21655-21662.
36. Liang Y, Fotiadis D, Maeda T, Maeda A, Modzelewska A, Filipek S, Saperstein DA, Engel A, Palczewski K. Rhodopsin signaling and organization in heterozygote rhodopsin knockout mice. J Biol Chem. 2004; 279: 48189-48196.
37. Liu X, Bulgakov OV, Wen XH, Woodruff ML, Pawlyk B, Yang J, Fain GL, Sandberg MA, Makino CL, Li T. AIPL1, the protein that is defective in Leber congenital amaurosis, is essential for the biosynthesis of retinal rod cGMP phosphodiesterase. Proc Natl Acad Sci USA. 2004; 101: 13903-13908.
38. Lyubarsky AL, Daniele LL, Pugh EN Jr. From candelas to photoisomerizations in the mouse eye by rhodopsin bleaching in situ and the light-rearing dependence of the major components of the mouse ERG. Vision Res. 2004; 44: 3235-3251.
39. Makino CL, Dodd RL, Chen J, Burns ME, Roca A, Simon MI, Baylor DA. Recoverin regulates light-dependent phosphodiesterase activity in retinal rods. J Gen Physiol. 2004; 123: 729-741.
40. Makino CL, Peshenko IV, Wen XH, Olshevskaya EV, Barrett R, Dizhoor AM. A role for GCAP2 in regulating the photoresponse. Guanylyl cyclase activation and rod electrophysiology in GUCA1B knock-out mice. J Biol Chem. 2008; 283: 29135-29143.
41. Makino CL, Wen XH, Michaud N, Peshenko IV, Pawlyk B, Brush RS, Soloviev M, Liu X, Woodruff ML, Calvert PD, Savchenko AB, Anderson RE, Fain GL, Li T, Sandberg MA, Dizhoor AM. Effects of low AIPL1 expression on phototransduction in rods. Invest Ophthalmol Vis Sci. 2006; 47: 2185-2194.
42. Makino CL, Wen XH, Olshevskaya EV, Peshenko IV, Savchenko AB, Dizhoor AM. Enzymatic relay mechanism stimulates cyclic GMP synthesis in rod photoresponse: biochemical and physiological study in guanylyl cyclase activating protein 1 knockout mice. PLoS ONE. 2012; 7: e47637. doi: 10.1371/journal.pone.0047637.
43. Mariani AP. Photoreceptors of the larval tiger salamander retina. Proc R Soc Lond B Biol Sci. 1986; 227: 483-492.
44. Mendez A, Burns ME, Roca A, Lem J, Wu LW, Simon MI, Baylor DA, Chen J. Rapid and reproducible deactivation of rhodopsin requires multiple phosphorylation sites. Neuron. 2000; 28: 153-164.
45. Mendez A, Chen J. Mouse models to study GCAP functions in intact photoreceptors. Adv Exp Med Biol. 2002; 514: 361-388.
46. Nakatani K, Chen C, Koutalos Y. Calcium diffusion coefficient in rod photoreceptor outer segments. Biophys J. 2002; 82: 728-739.
47. Nikonov S, Engheta N, Pugh EN Jr. Kinetics of recovery of the dark-adapted salamander rod photoresponse. J Gen Physiol. 1998; 111: 7-37.
48. Nikonov S, Lamb TD, Pugh EN Jr. The role of steady phosphodiesterase activity in the kinetics and

sensitivity of the light-adapted salamander rod photoresponse. J Gen Physiol. 2000; 116: 795-824.
49. Olson A, Pugh EN Jr. Diffusion coefficient of cyclic GMP in salamander rod outer segments estimated with two fluorescent probes. Biophys J. 1993; 65: 1335-1352.
50. Papermaster DS, Reilly P, Schneider BG. Cone lamellae and red and green rod outer segment disks contain a large intrinsic membrane protein on their margins: an ultrastructural immunocytochemical study of frog retinas. Vision Res. 1982; 22: 1417-1428.
51. Pugh EN Jr, Duda T, Sitaramayya A, Sharma RK. Photoreceptor guanylate cyclases: a review. Biosci Rep. 1997; 17: 429-473.
52. Pugh EN Jr, Lamb TD. Cyclic GMP and calcium: the internal messengers of excitation and adaptation in vertebrate photoreceptors. Vision Res. 1990; 30: 1923-1948.
53. Pugh EN Jr, Lamb TD. Amplification and kinetics of the activation steps in phototransduction. Biochim Biophys Acta. 1993; 1141: 111-149.
54. Pugh EN Jr, Lamb TD. Phototransduction in vertebrate rods and cones: Molecular mechanisms of amplification, recovery and light adaptation. In Stavenga DG, DeGrip WJ, Pugh EN Jr, editors. Handbook of Biological Physics volume 3 Molecular mechanisms in visual transduction. Amsterdam, Elsevier Science BV; 2000. pp. 183-255.
55. Qin N, Baehr W. Expression and mutagenesis of mouse rod photoreceptor cGMP phosphodiesterase. J Biol Chem. 1994; 269: 3265-3271.
56. Ramanathan S, Detwiler PB, Sengupta AM, Shraiman BI. G-protein-coupled enzyme cascades have intrinsic properties that improve signal localization and fidelity. Biophys J. 2005; 88: 3063-3071.
57. Reiländer H, Achilles A, Friedel U, Maul G, Lottspeich F, Cook NJ. Primary structure and functional expression of the Na/Ca,K-exchanger from bovine rod photoreceptors. EMBO J. 1992; 11: 1689-1695.
58. Roof D, Adamian M, Jacobs D, Hayes A. Cytoskeletal specializations at the rod photoreceptor distal tip. J Comp Neurol. 1991; 305: 289-303.
59. Sampath AP, Matthews HR, Cornwall MC, Fain GL. Bleached pigment produces a maintained decrease in outer segment Ca^{2+} in salamander rods. J Gen Physiol. 1998; 111: 53-64.
60. Schnetkamp PP. Na-Ca or Na-Ca-K exchange in rod photoreceptors. Prog Biophys Mol Biol. 1989; 54: 1-29.
61. Schnetkamp PP. Cation selectivity of and cation binding to the cGMP-dependent channel in bovine rod outer segment membranes. J Gen Physiol. 1990; 96: 517-534.
62. Schnetkamp PP. Optical measurements of Na-Ca-K exchange currents in intact outer segments isolated from bovine retinal rods. J Gen Physiol. 1991; 98: 555-573.
63. Schnetkamp PP, Szerencsei RT, Basu DK. Unidirectional Na^+, Ca^{2+}, and K^+ fluxes through the bovine rod outer segment Na-Ca-K exchanger. J Biol Chem. 1991; 266: 198-206.
64. Shen L, Caruso G, Bisegna P, Andreucci D, Gurevich VV, Hamm HE, DiBenedetto E. Dynamics of mouse rod phototransduction and its sensitivity to variation of key parameters. IET Syst Biol.

2010; 4: 12-32.
65. Sherry DM, Bui DD, DeGrip WJ. Identification and distribution of photoreceptor subtypes in the neotenic salamander retina. Vis Neurosci. 1998; 15: 1175-1187.
66. Sitaramayya A, Harkness J, Parkes JH, Gonzalez-Oliva C, Liebman PA. Kinetic studies suggest that light-activated cyclic GMP phosphodiesterase is a complex with G-protein subunits. Biochemistry. 1986; 25: 651-656.
67. Tsang SH, Burns ME, Calvert PD, Gouras P, Baylor DA, Goff SP, Arshavsky VY. Role for the target enzyme in deactivation of photoreceptor G protein in vivo. Science. 1998; 282: 117-121.
68. Tsukamoto Y. The number, depth and elongation of disc incisures in the retinal rod of *Rana catesbeiana*. Exp Eye Res. 1987; 45: 105-116.
69. Wang Z, Wen XH, Ablonczy Z, Crouch RK, Makino CL, Lem J. Enhanced shutoff of phototransduction in transgenic mice expressing palmitoylation-deficient rhodopsin. J Biol Chem. 2005; 280: 24293-24300.
70. Wilden U. Duration and amplitude of the light-induced cGMP hydrolysis in vertebrate photoreceptors are regulated by multiple phosphorylation of rhodopsin and by arrestin binding. Biochemistry. 1995; 34: 1446-1454.
71. Woodruff ML, Lem J, Fain GL. Early receptor current of wild-type and transducin knockout mice: photosensitivity and light-induced Ca^{2+} release. J Physiol (Lond). 2004; 557: 821-828.
72. Woodruff ML, Sampath AP, Matthews HR, Krasnoperova NV, Lem J, Fain GL. Measurement of cytoplasmic calcium concentration in the rods of wild-type and transducin knock-out mice. J Physiol (Lond). 2002; 542: 843-854.
73. Xu J, Dodd RL, Makino CL, Simon MI, Baylor DA, Chen J. Prolonged photoresponses in transgenic mouse rods lacking arrestin. Nature. 1997; 389: 505-509.
