# Supplementary material for: Position of rhodopsin photoisomerization on the disk surface confers variability to the rising phase of the single photon response in vertebrate rod photoreceptors
Source: PLoS One. 2020 Oct 14;15(10):e0240527. doi: 10.1371/journal.pone.0240527 (PMC7556485; doi:10.1371/journal.pone.0240527)
Supplement: S1 File — (PDF) [file pone.0240527.s002.pdf]

# S1 File. Simulations in which two transducins are necessary for PDE activation.

Position of rhodopsin photoisomerization on the disk surface confers variability to the rising phase of the single photon response in vertebrate rod photoreceptors

Giovanni Caruso
Italian National Research Council, Istituto di Scienze del Patrimonio Culturale
Via Salaria Km. 29,300- C.P. 10 Monterotondo St., Roma, Italy
email: giovanni.caruso@itabc.cnr.it

Colin J. Klaus
The Mathematical Biosciences Institute, Ohio State University,
1735 Neil Avenue, Columbus, OH 43210
email: klaus.68@mbi.osu.edu

Heidi E. Hamm
Department of Pharmacology, Vanderbilt University Medical Center,
2200 Pierce Avenue, Preston Research Building Rm 452
Nashville, TN 37232
email: heidi.hamm@vanderbilt.edu

Vsevolod V. Gurevich
Department of Pharmacology, Vanderbilt University Medical Center,
2200 Pierce Avenue, Preston Research Building Rm 452
Nashville, TN 37232
email: Vsevolod.Gurevich@vanderbilt.edu

Clint L. Makino
Department of Physiology and Biophysics, Boston University School of Medicine
W402 700 Albany Street, Boston, MA 02118-2526
email: cmakino@bu.edu

Emmanuele DiBenedetto
Department of Mathematics, Vanderbilt University
1326 Stevenson Center, Nashville, TN 37240
email: em.diben@vanderbilt.edu

The model herein implements 2T*:PDE** rather than T*:PDE* by modifying the mass action term of **S1 Appendix,** section A4. In both equations there, $k_{T^*E}$[E][T*] is replaced with $k_{T^*E}$[E][T*]$^2$. Parameter changes were limited to substitutions in $k_{T^*E}$ and $\nu_{RG}$. There were minor changes in the resultant SPR (cf. **Fig S3** in **S1 File**). No attempt was made to vary other parameters so that the response would more closely match the experimentally observed SPR , because the purpose of the modeling was to find out whether there would be substantial effects on the radial gradients of cGMP.

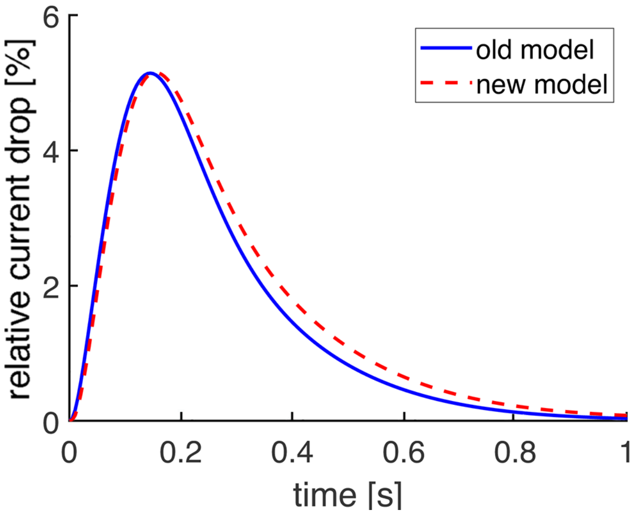


**Fig S3**. **Single photon response in mouse.** Disks each had 1 incisure. $D_{cG}$ = 120 $\mu$m$^2$ s$^{-1}$, $k_{T^*E}$ = 0.01 $\mu$m$^2$ s$^{-1}$, $\nu_{RG}$ = 357 s$^{-1}$.

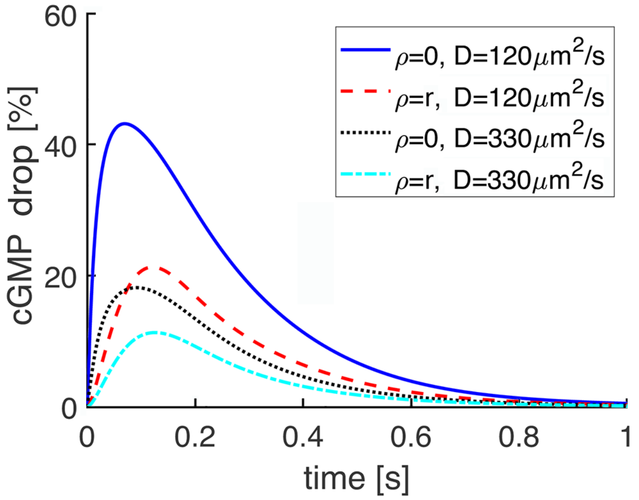

**Fig S4. Radial gradients in cGMP for an R* located in the center of a mouse disk for two values of $D_{cG}$.** Disks lacked the incisure. Disk radius was 0.685 $\mu$m. For $D_{cG}$ = 120 $\mu m^2\ s^{-1}$: $k_{T^*E}$ = 0.01 $\mu m^2\ s^{-1}$, $\nu_{RG}$ = 357 $s^{-1}$, while for $D_{cG}$ = 330 $\mu m^2\ s^{-1}$: $k_{T^*E}$ = 0.01 $\mu m^2\ s^{-1}$, $\nu_{RG}$ = 264 $s^{-1}$.

With PDE activation requiring two transducins, transverse gradients of cGMP were still generated during the single photon response (**Fig S4**). There were subtle differences; compared to the gradients obtained with PDE subunit activation by single transducins (**Fig 6**), the maxima were slightly lower and the difference between the drop at disk center versus at the disk rim were reduced by about 10%. Furthermore, the gradients took slightly longer to dissipate than before. However, there were no significant changes in the CV profiles (**Fig S5**, cf. **Fig 7B,D**), so the conclusion is that the nature of PDE activation does not affect the variability in SPR produced by the location of R* on the disk surface.

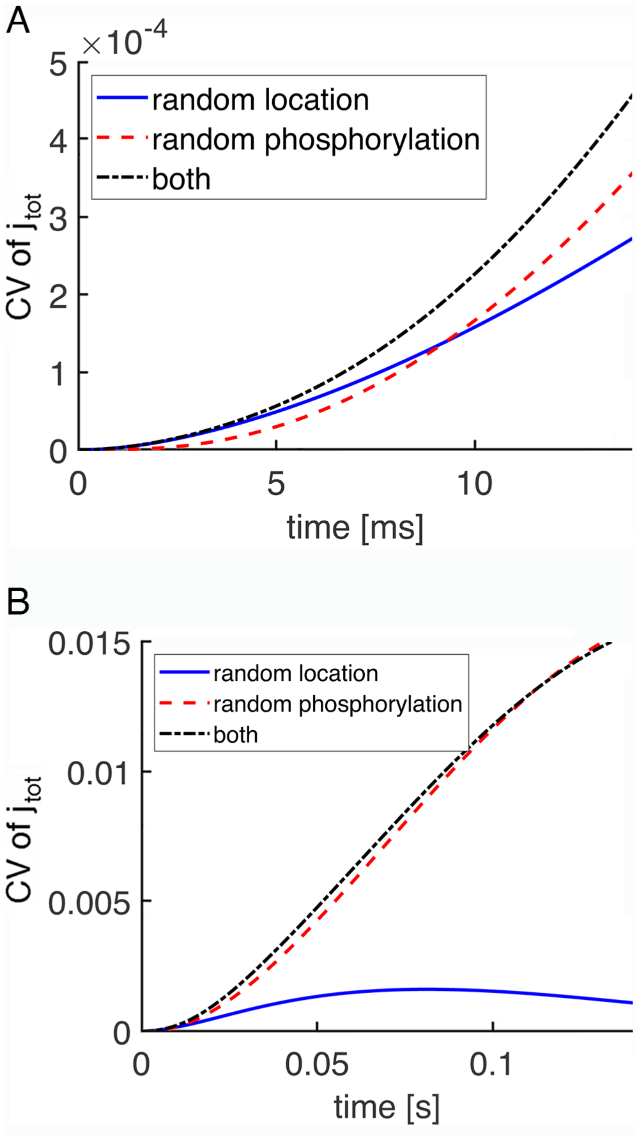

**Fig S5. CV of the total current for the SPR in mouse.** CV arising from the random location of R* was greater than that due to random phosphorylation during the first few ms of the SPR (**A**), but thereafter (**B**), randomness in R* shutoff was the main source of variability. Each disk had an incisure. Disk radius was 0.685 $\mu$m. $D_{cG} = 120\ \mu m^2\ s^{-1}$, $k_{T^*E} = 0.01\ \mu m^2\ s^{-1}$, and $\nu_{RG} = 357\ s^{-1}$.
